# Cryopreservation of human cerebral microvascular endothelial cells and astrocytes in suspension and monolayers

**DOI:** 10.1371/journal.pone.0249814

**Published:** 2021-04-14

**Authors:** Leah A. Marquez-Curtis, Reid Bokenfohr, Locksley E. McGann, Janet A. W. Elliott

**Affiliations:** 1 Department of Chemical and Materials Engineering, University of Alberta, Edmonton, AB, Canada; 2 Department of Laboratory Medicine and Pathology, University of Alberta, Edmonton, AB, Canada; 3 Department of Medicine, University of Alberta, Edmonton, AB, Canada; University of Maryland, UNITED STATES

## Abstract

The blood–brain barrier (BBB) keeps pathogens and toxins out of the brain but also impedes the entry of pharmaceuticals. Human cerebral microvascular endothelial cells (hCMECs) and astrocytes are the main functional cell components of the BBB. Although available commercially as cryopreserved cells in suspension, improvements in their cryopreservation and distribution as cryopreserved monolayers could enhance BBB *in vitro* studies. Here, we examined the response to slow cooling and storage in liquid nitrogen of immortalized hCMEC/D3 cells and human primary astrocytes in suspension and in monolayers. HCMEC/D3 cells in suspension cryopreserved in 5% dimethyl sulfoxide (DMSO) and 95% fetal bovine serum or in 5% DMSO and 6% hydroxyethyl starch (HES) showed post-thaw membrane integrities above 90%, similar to unfrozen control. Cryopreservation did not affect the time-dependent ability of hCMEC/D3 cells to form tubes on Matrigel. Primary astrocytes in suspension cryopreserved in the presence of 5% DMSO and 6% HES had improved viability over those cryopreserved in 10% DMSO. Monolayers of single cultures or co-cultures of hCMEC/D3 cells and astrocytes on fibronectin-coated Rinzl coverslips retained membrane integrities and metabolic function, after freezing in 5% DMSO, 6% HES, and 2% chondroitin sulfate, that were comparable to those of unfrozen controls even after overnight incubation. Rinzl is better than glass or Thermanox as an underlying solid substrate for cryopreserving hCMEC/D3 monolayers. Cryopreserved hCMEC/D3 monolayers expressed the junction proteins ZO-1 and claudin-5 similar to their unfrozen counterparts. Hence, we describe improved cryopreservation protocols for hCMEC/D3 cells and astrocytes in suspension, and a novel protocol for the cryopreservation of monolayers of hCMEC/D3 cells and astrocytes as single cultures or co-cultures that could expand their distribution for research on disease modeling, drug screening, and targeted therapy pertaining to the BBB.

## Introduction

The blood–brain barrier (BBB) regulates the passage of soluble and cellular substances from the blood into the central nervous system (CNS) [1]. The blood vessels of the BBB are composed of continuous endothelial cells which are joined together by intracellular tight junctions and lack pores in their plasma membranes [2]. The BBB is a dynamically regulated partition that is permeable but highly selective, allowing the entry of small essential molecules like oxygen, amino acids, and glucose, but preventing the passage of pathogens and toxins [1, 3]. Impairment of the BBB has been associated with several pathologies such as Alzheimer’s disease, amyotrophic lateral sclerosis, epilepsy, stroke, and multiple sclerosis, and infections such as meningitis, syphilis, and HIV [4, 5]. However, the same barrier that protects the CNS from harm becomes an impediment for efficient delivery of neurological drugs and other pharmaceuticals [1, 4]. Research on diseases of the CNS, drug permeability testing, BBB toxicity, and drug target studies requires an *in vitro* model of the BBB that can be available on demand. Cryopreservation is the technology that can make this possible. Because the interactions of microvascular endothelial cells with astrocytes are necessary for the formation, maintenance, and regulation of the BBB [1, 5], this work describes the cryopreservation of these cells in suspension, and in monolayers as single cultures and co-cultures.

The immortalized human cerebral microvascular endothelial cell (hCMEC)/D3 line [6] is one of the most extensively characterized and widely used *in vitro* models of the human BBB for drug transport studies [7–12]. This cell line retains the expression of most transporters, receptors, and tight junction proteins expressed *in vivo* by the human BBB (such as zonula occludens, for example ZO-1, and claudin-5), and demonstrates several key functional properties of vascular endothelium such as angiogenic tube formation [2, 8, 9]. Therefore, refinement of cryopreservation of this cell line could present a great benefit for research pertaining to the BBB. One drawback for most immortalized human cell lines used for BBB research is that the immortalization process inherently changes the immune response [6, 13, 14]. Furthermore, in studies of amyloid beta oligomer transport, hCMEC/D3 cells have been shown to have lower trans-endothelial electrical resistance (TEER), a measurement associated with tight junction formation [15]. However, co-culturing hCMEC/D3 cells with astrocytes increases TEER values, and induces a phenotype comparable to primary brain microvascular endothelial cells [7, 10, 15–22]. Therefore, in addition to cells in suspension, cryopreservation of these cells as single cultures and co-cultures in a monolayer configuration will provide *in vitro* models of the BBB on demand.

First, we examined the cryobiological responses of hCMEC/D3 cells and astrocytes in suspension slowly cooled below the freezing point of the solution. As ice forms outside the cells, the extracellular solute concentration increases and the cells respond by releasing water. Prolonged exposure of cells to this non-physiological state at moderate sub-zero temperatures causes what is referred to as “solute effects” or slow-cooling injury [23]. On the other hand, cells that are cooled too rapidly are susceptible to intracellular ice formation, which is lethal to cells in suspension. In addition, intracellular ice may recrystallize during slow thawing, causing further cellular damage [23–25]. Cryoprotective agents (CPAs) mitigate these injuries [26]. Penetrating CPAs cross the plasma membrane, increase intracellular and extracellular osmolality, reduce the amount of ice formed via freezing point depression, and protect cells from excessive dehydration. Dimethyl sulfoxide (DMSO) is a well-characterized, water soluble, penetrating CPA [27–29]. Non-penetrating CPAs, such as hydroxyethyl starch (HES), are unable to cross intact plasma membranes and therefore increase extracellular osmolality, drawing water out of the cells earlier in the cooling profile and reducing the likelihood of intracellular ice formation [26, 30–32]. Although CPAs increase cell survival following cryopreservation, they can also reduce cell viability via direct toxic effects, indirect toxicity via osmotic effects, or during the process of removing them from the thawed cells [33, 34]. Thus, in order to maximize cell viability after cryopreservation, lower concentrations of CPAs, as well as lower temperature and shorter exposure are appropriate. We employed an interrupted slow cooling procedure (also called graded freezing) to identify the occurrence and extent of cryoinjury, and to examine the protection conferred by penetrating and non-penetrating CPAs [35–38]. We previously demonstrated that the combination of 5% DMSO and 6% HES resulted in the highest post-thaw membrane integrity (87.7 ± 0.8%) of human umbilical vein endothelial cells (HUVECs), and human corneal endothelial cells (89.1 ± 0.6%) in suspension [37, 38]. In addition to membrane integrity assessment, it is important to establish post-thaw functional activity of hCMEC/D3 cells in suspension, in this case their ability to form three dimensional capillary-like networks [39] in Matrigel, a commercially available substrate containing angiogenic growth factors [40]. In this work, we investigate potential improvements upon supplier-recommended cryopreservation protocols for hCMEC/D3 cells and astrocytes in suspension.

Second, we cryopreserved hCMEC/D3 and astrocyte monolayers whose two-dimensional configuration more closely recapitulates *in vivo* interactions. The cryopreservation of monolayers presents additional challenges over cells in suspension because of cell contacts. The presence of cell–cell junctions and cell–surface (underlying solid substrate or matrix) interactions in the monolayer format increases the likelihood of intracellular ice formation compared to cells in suspension [41–44]. For cell monolayers, the properties of the substrate/matrix surface on which the cells are cultured may also affect cell attachment and survival during the freeze-thaw process. Previously we showed that fibroblast monolayers grown on Rinzl plastic, whose coefficient of linear thermal expansion (*α*_*L*_, 60 × 10^−6^/K), is similar to ice (51 × 10^−6^/K), had better adhesion after freezing and thawing over those cultured on glass, whose *α*_*L*_ is lower (5 × 10^−6^/K) than ice [45]. Moreover, coating Rinzl with fibronectin, adding 2% chondroitin sulfate to 5% DMSO and 6% HES, controlling ice nucleation, and cooling cells slowly before plunging into liquid nitrogen resulted in high viability of HUVECs and porcine corneal endothelial monolayers immediately after thaw and after post-thaw overnight incubation [46]. In this work, in order to isolate the effect of the substrate from the effect of the cryopreservation procedure, we compared the cryobiological response of hCMEC/D3 monolayers cultured on Rinzl (*α*_*L*_ matched to ice) to those cultured on glass (lower *α*_*L*_ than ice) and on Thermanox (higher *α*_*L*_ (124 x 10^−6^/K [47]) than ice). We then applied our optimized cryopreservation protocol [46, 48] to hCMEC/D3 and astrocyte monolayers in single cultures and co-cultures. Since it has been shown that cryopreservation-induced cell death may manifest itself within 6–48 hours after thaw [49], we also investigated whether cryopreserved monolayers of hCMEC/D3 and astrocyte single cultures and co-cultures retain their viability after overnight incubation post-thaw. In addition to retention of membrane integrity and metabolic activity of cryopreserved single cultures and co-cultures, we further examined the expression of tight junction proteins ZO-1 and claudin-5 in cryopreserved hCMEC/D3 monolayers.

## Materials and methods

### Cultures of hCMEC/D3 cells and human astrocytes

The human cerebral microvascular endothelial cell/D3 clone (hCMEC/D3 cell line, CLU512, Cedarlane, CELLutions Biosystems Inc., Burlington, ON, Canada) was purchased as cryopreserved cells in 5% DMSO and 95% fetal bovine serum (FBS) [50]. The cells were received on dry ice, immediately transferred to liquid nitrogen upon delivery, and rapidly thawed in a 37°C water bath prior to plating. The hCMEC/D3 cells were cultured in endothelial basal medium (EBM, 190860, Lonza Group Ltd., Walkersville, MD, USA) supplemented with 5% FBS (10270–106, Life Technologies, Grand Island, NY, USA), 1.4 μM hydrocortisone (H0135, Millipore Sigma, Burlington, MA, USA), 5 μg/mL ascorbic acid (A4544, Millipore Sigma), 1% chemically defined lipid concentrate (111905031, Life Technologies), 1% penicillin/streptomycin (Life Technologies), 10 mM HEPES (15630–080, Life Technologies) and 1 ng/mL basic fibroblast growth factor (F0291, Millipore Sigma), on Falcon flasks (353135, Thermo Fisher Scientific, Ottawa, ON, Canada) treated with Cultrex rat collagen I (3443-100-01, Trevigen, R&D Systems, Minneapolis, MN, USA) or CellBIND flasks (CLS3290, Corning Life Sciences, Tewksbury, MA, USA) in a humidified incubator at 37°C and 5% CO_2_. The medium was changed every 3 to 4 days until the cells reached ~80% confluence. The hCMEC/D3 cells were harvested by addition of 0.25% trypsin-EDTA (IX, 25200, Gibco, Thermo Fisher Scientific) and incubation at 37°C for 2–3 min. Trypsinization was stopped by adding serum-containing medium, followed by centrifugation at 13640 x g for 10 min in an Eppendorf 5810R tabletop centrifuge (Eppendorf AG, Hamburg, Germany). The cell pellet was resuspended in medium and counted using a Coulter® Z2^TM^ particle count and size analyzer (Beckman Coulter, Mississauga, ON, Canada). HCMEC/D3 cells were used in graded freezing of cell suspensions, sub-cultured in flasks at 25,000 cells per cm^2^, or seeded onto fibronectin-covered coverslips for monolayer experiments. Comparison of cell population doubling time was made between Falcon flasks pre-treated at 37°C for at least 1 h with 6 mL of 150 μg/mL rat collagen I *vs*. untreated Corning CellBIND surface cell culture flasks (US Patent 6,617,152) [51]. The doubling time was defined as:

$$Doubling\;time=\frac{t-t_{0}}{\log_{2}\left(\frac{C_{final}}{C_{0}}\right)}$$ (1)

where: *t* = final time, *t*_0_ = initial time, *C*_final_ = final cell concentration, and *C*_0_ = initial cell concentration.

Primary human astrocytes from normal brain tissue (N7805-100, K1884, Gibco, Thermo Fisher Scientific), cryopreserved in 10% DMSO, were received on dry ice and immediately placed in liquid nitrogen. Before thawing and plating the cells, it was necessary to pre-coat tissue culture flasks with Geltrex basement membrane matrix (A14132, Thermo Fisher). Geltrex was first diluted 1:1 with Dulbecco’s Modified Eagle Medium (DMEM, 10569–010, Gibco) and stored in stock aliquots at –20°C until needed. The stock solution was further diluted 1:100 with DMEM, and 200 μL was used per cm^2^ of culture surface. The culture vessel was incubated for 1 h at 37°C, the Geltrex solution was aspirated, and the culture surface rinsed with Dulbecco’s phosphate-buffered saline (DPBS) containing Ca^2+^ and Mg^2+^ (14040133, Thermo Fisher) before cell seeding. Because astrocytes readily stick to plastic, culture surfaces must be pre-wet with complete astrocyte medium before adding the cell suspension. The complete medium was composed of 89 mL DMEM, 1 mL N2 Supplement (100X, 17502–048, Thermo Fisher) and 10 mL FBS. Cells were thawed by gentle agitation in a 37°C water bath, and transferred to a pre-wet 15-mL centrifuge tube using a sterile pipette tip previously wet with medium. The cells were suspended in 5 mL of medium, and then centrifuged at 290 × g for 5 min. The cell pellet was resuspended in medium and seeded at 4 × 10^4^ cells/cm^2^ (e.g., 1 × 10^6^ cells in 5 mL complete medium in a T25 flask). The cells were incubated at 37°C in a humidified atmosphere of 5% CO_2_ in air and the medium changed every 2 days until ~80% confluence. The cell-conditioned medium was collected and used to stop the action of cell dissociation enzyme (StemPro® Accutase® Cell Dissociation Reagent, A11105, Thermo Fisher). The cells were washed once with 1X DPBS without calcium, magnesium, or phenol red (14190250, Thermo Fisher). Accutase® was added at 10 mL per 75 cm^2^ surface area, and then the cells were incubated for 5–10 min at 37°C. An equal volume (1:1) of conditioned medium was added to stop the Accutase® activity and then the cells were centrifuged for 5 min at 290 × g. The cell pellet was resuspended in 2–3 mL complete astrocyte medium, counted, then used in graded freezing of cell suspensions, sub-cultured at 4 × 10^4^ cells/cm^2^ on Geltrex-treated flasks, or seeded onto fibronectin-covered Rinzl coverslips for monolayer experiments.

### Preparation of cryoprotectants

Cryoprotectant stock solutions were prepared by weight as: *i*) 10% DMSO (Fisher Scientific, Edmonton, AB, Canada) in FBS; *ii*) 20% DMSO in medium; *iii*) 10% DMSO plus 12% hydroxyethyl starch (HES, PST002, 20% Pentastarch Solution, Preservation Solutions Inc., Elkhorn, WI, USA) in medium; and *iv*) 10% DMSO plus 12% HES plus 4% chondroitin sulfate (CS, C9819, Sigma) in medium. For experiments on cells in suspension, equal weight of cryoprotectant solution was added to resuspended cells to attain final CPA concentrations of: *i*) 5% (w/w) DMSO in FBS; *ii*) 10% (w/w) DMSO in medium; and *iii*) 5% (w/w) DMSO plus 6% (w/w) HES in medium. For experiments on monolayers, equal volume (95 μL) of medium was added to cryoprotectant solution to attain a final concentration of 5% DMSO, 6% HES, and 2% CS.

### Graded freezing of cells in suspension

Interrupted slow cooling, also called graded freezing, was carried out as previously described [36–38, 52]. Aliquots of 200 μL of hCMEC/D3cells or astrocytes suspended in their respective medium, in the presence or absence of cryoprotectant, were transferred into 6 × 50 mm borosilicate glass test tubes (VWR, Edmonton, AB, Canada). The cell suspensions in the presence of CPAs were kept on ice for 15 min (this period of time allows the DMSO to permeate the cells), and then equilibrated for 2 min in a stirred methanol bath (FTS Systems Inc., Stone Ridge, NY, USA) set at −5°C. For each experimental group, one pair of test tubes was plunged directly into liquid nitrogen (dead control) and another pair was used as live, unfrozen control. For the rest of the tubes, following equilibration at −5°C ice nucleation was induced using metal forceps cooled in liquid nitrogen. The test tubes were left in the methanol bath for 3 min to allow the release of the latent heat of fusion after which the methanol bath was set to cool at 1°C/min to –40°C. The temperature was monitored using a T-type thermocouple and OMB-DAQ-55 data acquisition module and Personal Daq View software (OMEGA Engineering Inc., Stamford, CT, USA) after calibrating to an ice–water standard. Four test tubes were removed at –10°C, –20°C, –30°C, and –40°C. Two test tubes were directly thawed (DT group) by rapid warming in a 37°C water bath, while the other two tubes were plunged directly into liquid nitrogen and left for at least an hour before rapid warming in a 37°C water bath (plunge-thaw (PT) group). After thawing, cells were immediately stained for membrane integrity assessment, and in the case of hCMEC/D3 cells cultured for tube formation and immunocytochemistry, as described below.

### Cryopreservation of cell monolayers

The hCMEC/D3 cells were sub-cultured on three different underlying solid substrates namely glass, Rinzl (clear vinyl plastic, 72261–18) and Thermanox (clear polyolefin plastic, 72270) coverslips. The plastic coverslips (both from Electron Microscopy Sciences, Hatfield, PA, USA) were cut to approximately 9 x 9 mm squares, sterilized by immersing in 70% ethanol for at least 30 min, then washed with phosphate-buffered saline (1X PBS, Life Technologies) for 15 min before transfer to a 24-well plate (Cellstar®, 662160, Greiner Bio-One, Monroe, NC, USA). To promote cell attachment, the coverslips were pre-treated with fibronectin from bovine plasma (F-1141, Sigma-Aldrich, Oakville, ON, Canada) at a concentration of 2.5 μg/mL for at least 30 min at room temperature. The fibronectin solution was removed and the coated coverslips were used without washing. HCMEC/D3 cells were seeded at a density of at least 1 × 10^4^ cells/cm^2^ on fibronectin-coated coverslips in 500 μL of medium. The medium was replaced every other day until confluence was attained. Coverslips (with the cell monolayer side up) were transferred to glass vials (45×15mm, 60965D-1, Kimble Chase, Rochester, NY, USA) containing 190 μL of medium with CPA (5% DMSO + 6% HES + 2% CS). The cell monolayers were incubated for 15 min on ice, equilibrated at –5°C in a stirred methanol bath for 2 min, ice nucleated (by touching the vials using metal forceps pre-cooled in liquid nitrogen), and placed back in the methanol bath for 3 min to release the latent heat of fusion. The monolayers were then cooled at 1°C/min to –40°C and then plunged into liquid nitrogen, kept there for an hour, and then thawed in a 37°C water bath. The samples were assessed for membrane integrity as described below. Astrocytes were cultured on fibronectin-coated Rinzl plastic coverslips as single cultures and as co-cultures with hCMEC/D3 cells. The cells were seeded at a density of at least 1 × 10^4^ cells/cm^2^ in 500 μL of astrocyte medium for single cultures. In co-cultures, the cell density of each of hCMEC/D3 cells and astrocytes was 1 × 10^4^ cells/cm^2^ seeded in a combination of 250 μL of hCMEC/D3 medium and 250 μL of astrocyte medium.

### Membrane integrity assessment of cells in suspension by flow cytometry

Direct and plunge thawed cells in suspension were immediately analyzed by flow cytometry using a dual fluorescent stain composed of SYTO 13 (S7575, Molecular Probes, Eugene, OR, USA) and GelRed (41003, Biotium, Scarborough, ON, USA) as previously described [53]. The stain was prepared fresh on the day of the experiment from stock solutions of SYTO 13 (six μL of 5 mM), GelRed (10 μL of 10,000x in water) and 112 μL of distilled water. The hCMEC/D3 cells were stained following a “wash” step in order to remove FBS that produces background fluorescence, which interfered with flow cytometric analysis. Cell suspensions were centrifuged at 200 x g for 5 min followed by aspiration of the supernatant and resuspension of the pellet in 400 μL 1X DPBS. Twenty μL of stain was added to each sample; the samples were incubated for 10 min at room temperature in darkness prior to data acquisition using an Epics XL-MCL flow cytometer (Beckman Coulter Inc., Pasadena, CA, USA). The settings are described in detail elsewhere [52, 53]. Each event appeared as a dot on a scatter plot of GelRed fluorescence (red) versus SYTO 13 fluorescence (green). SYTO 13 penetrates the cell membranes of all cells and complexes with nucleic acids to emit a green fluorescence when excited by ultraviolet light [54]. GelRed penetrates only cells with damaged membranes and emits an intense red fluorescence. When cells stain positive for GelRed the SYTO 13 signal is quenched through a variety of mechanisms [54, 55]. Therefore, green fluorescing cells were considered to have intact membranes and consequently to be viable, while red fluorescing and doubly stained cells were considered to have damaged membranes and counted as non-viable. Flow cytometry results were analyzed using the Kaluza Analysis software (version 1.3) from Beckman-Coulter. The percent membrane integrity was calculated from the ratio of the number of membrane-intact cells (counted from the green quadrant) to the total number of cells (counted from the green, red, and double-stained quadrants).

$$\%\;Membrane\;integrity=\frac{\left(green\;fluorescing\;cells\right)}{\left(total\;cell\;count\right)}\times 100\%$$ (2)

### Membrane integrity and absolute viability assessments of cell monolayers by fluorescent microscopy

Membrane integrity assessment by fluorescent microscopy was carried out as previously described [46]. Because the chondroitin sulfate in the CPA cocktail masks the fluorescence signal, 500 μL of 1X PBS was added to the vial containing the cell monolayer after thaw, and the solution was removed after 5 min. 190 μL of PBS was then added to the vial and cells were stained with SYTO 13/GelRed. The stain (10 μL 10,000x GelRed in water plus 10 μL 5 mM SYTO 13 plus 262.5 μL of PBS) was prepared fresh and kept on ice in the dark. Ten μL of staining solution was added to each vial followed by incubation for 5 min in the dark. Coverslips were then transferred (cell side down) onto a glass slide and observed under a fluorescent microscope (Leitz, Dialux 22) at 10X magnification. Images were captured with an Infinity3 camera and Infinity Capture software (Lumenera Corporation, Ottawa, ON, Canada). At least 12 images were captured for each coverslip and membrane integrity in each captured image was quantified using the Viability3 program for automated cell counting (custom software version 3.2). The Viability3 program gives information about the total number of cells, numbers of green and red cells, and numbers of green and red pixels in each image. The percent membrane integrity and absolute viability were calculated using the following equations:

$$\%Membrane\;integrity=\frac{\;\mathrm{number}\;\mathrm{of}\;\mathrm{green}\;\mathrm{cells}\;\mathrm{on}\;a\;\mathrm{coverslip}\;}{\;\mathrm{total}\;\mathrm{number}\;\mathrm{of}\;\mathrm{cells}\;\mathrm{on}\;a\;\mathrm{coverslip}\;}\times 100\%$$ (3)

$$\%Absolute\;viability=\frac{\;\mathrm{number}\;\mathrm{of}\;\mathrm{green}\;\mathrm{cells}\;\mathrm{on}\;\mathrm{an}\;\mathrm{experimental}\;\mathrm{coverslip}\;}{\;\mathrm{total}\;\mathrm{number}\;\mathrm{of}\;\mathrm{cells}\;\mathrm{on}\;\mathrm{an}\;\mathrm{experimental}\;\mathrm{coverslip}\;}\times 100\%$$ (4)

### Assessment of membrane integrity and metabolic activity of monolayers after extended incubation

HCMEC/D3 cells and astrocytes were cultured to confluence separately or together (co-cultures) on fibronectin-coated coverslips. Coverslips (with the cell monolayer side up) were transferred to glass vials containing 190 μL of medium with CPAs (5% DMSO + 6% HES + 2% CS) and cryopreserved as described above. A set of monolayers were either immediately stained with SYTO 13/GelRed or assessed for metabolic activity by AlamarBlue reduction. Another set of thawed monolayers were incubated overnight (at least 16 h) in medium at 37°C and 5% CO_2_. The next day, assessments of membrane integrity (SYTO 13/GelRed staining) and AlamarBlue reduction were carried out. AlamarBlue is a reagent that contains the cell-permeable and non-toxic indicator dye called resazurin. Resazurin (oxidized form, blue) detects cell metabolic activity by converting to resorufin (reduced form, red) in response to chemical reduction of growth medium resulting from cell growth. The colorimetric signal detected at 570 nm is proportional to the number of cells with innate metabolic activity in the sample. Because there is considerable overlap in the absorption spectra of the oxidized and reduced forms of AlamarBlue, absorbance was measured at two wavelengths namely 570 nm and 600 nm. The percent reduction of AlamarBlue is calculated using the following equation [56]:

$$\%\;AlamarBlue\;reduction=\frac{{\left(\varepsilon_{OX}\right)}_{\lambda_{2}}A_{\lambda_{1}}-{\left(\varepsilon_{OX}\right)}_{\lambda_{1}}A_{\lambda_{2}}}{{\left(\varepsilon_{RED}\right)}_{\lambda_{1}}{A\prime }_{\lambda_{2}}-{\left(\varepsilon_{RED}\right)}_{\lambda_{2}}{A\prime }_{\lambda_{1}}}\times 100\%$$ (5)

where ε_OX_ is the molar extinction coefficient of AlamarBlue oxidized form (blue), ${\left(\varepsilon_{OX}\right)}_{\lambda_{1}=570\;nm}$ = 155,677, ${\left(\varepsilon_{OX}\right)}_{\lambda_{2}=600\;nm}$ = 14,652; ε_RED_ is the molar extinction coefficient of AlamarBlue reduced form (red), ${\left(\varepsilon_{RED}\right)}_{\lambda_{1}=570\;nm}$ = 80,586, ${\left(\varepsilon_{RED}\right)}_{\lambda_{2}=600\;nm}$ = 17,216; A is absorbance of test wells at *λ*_1_ = 570 nm and *λ*_2_ = 600 nm; and A′ is absorbance of negative control well (contains medium and AlamarBlue but no cells) at *λ*_1_ = 570 nm and *λ*_2_ = 600 nm.

Twenty μL of AlamarBlue reagent (Invitrogen, Life Technologies Inc., Eugene, OR, USA) was added to 200 μL of medium covering the monolayers and the samples were incubated for at least 4 h at 37°C. Absorbance was measured at 570 nm using 600 nm as a reference wavelength (SpectraMax Plus, Molecular Devices, San Jose, CA, USA). The percent reduction of AlamarBlue was calculated as per manufacturer’s instructions with corrections applied for no-cell controls (Eq 5).

### Immunocytochemistry

HCMEC/D3 cell monolayers were stained for the tight junction proteins ZO-1 (monoclonal antibody Alexa Fluor 488, 339188, Invitrogen) and claudin-5 (monoclonal antibody Alexa Fluor 488, 352588, Invitrogen) immediately after thaw as previously described [38]. Fresh and cryopreserved hCMEC/D3 cells were cultured on fibronectin-coated glass coverslips (12-mm diameter) placed in 24-well plates with 500 μL of medium. At confluence, the cells were fixed with either methanol cooled at –20°C for 20 min (claudin-5), or with 3.7% paraformaldehyde (PFA, Sigma) in PBS for 20 min at room temperature (ZO-1) and rinsed three times with PBS. Permeabilization of PFA-fixed cells was performed using 0.1% Triton X-100 (Fisher Scientific) for 10 min at room temperature followed by blocking with 3% bovine serum albumin (BSA, Sigma) for at least an hour in the dark at room temperature. Methanol-fixed cells did not need permeabilization; blocking was carried out using 10% goat serum for an hour at room temperature. Fluorochrome-conjugated antibodies (stock concentration: 0.5 mg/mL) were diluted in PBS containing 1% BSA and 1% goat serum (claudin-5 at 1:50 dilution) or in PBS containing 3% BSA and 0.3% Triton X-100 (ZO-1 at 1:100 dilution), and incubated with the cell monolayer overnight at 4°C. The antibody was removed, and the monolayer washed three times with PBS. The coverslips were mounted on glass slides and imaged using a fluorescent microscope (Leitz, Dialux 22).

### Matrigel tube formation assay

Matrigel basement membrane matrix (Corning, Bedford, MA, USA) was thawed from –20°C by leaving it overnight at 4°C and keeping on ice until use. A 75-μL aliquot was dispensed onto each well of a chilled 96-well culture plate using pre-cooled pipet tips. The plate was incubated at 37°C for 30–60 min to allow the Matrigel to solidify. Fresh unfrozen hCMEC/D3 cells (15,000 cells in 100 μL medium) were added to each well containing Matrigel. The plate was incubated at 37°C and 5% CO_2_, and tube formation was observed at 40X magnification at different time points using the Labovert phase contrast microscope (Leitz, Los Angeles, CA, USA). Images were captured with an attached Diractor camera (Pixera, Santa Clara, CA, USA), and the Angiogenesis Analyzer software was used to quantitate the tube length [57]. HCMEC/D3 cells in medium containing 5% DMSO and 6% HES were also cryopreserved as described above. The cryoprotectants in the samples were removed by single wash using PBS, followed by centrifugation and aspiration of the supernatant. The cell pellets were resuspended in medium, and 100 μL of the cell suspension (containing 15,000 cells) were added to each well containing Matrigel. The plate was incubated for 4 h at 37°C and 5% CO_2_. Tube formation was assessed as before.

### Statistical analysis

Statistical significance was defined at 95% confidence level and calculated using an unpaired, two-tailed, Student’s t-test. Statistical calculations were carried out using Microsoft Excel 2016 (Microsoft Corporation, Redmond, Washington, United States). All membrane integrity experiments were performed in triplicate.

## Results

### Growth kinetics and cryobiological response of hCMEC/D3 cells

**Fig 1:**
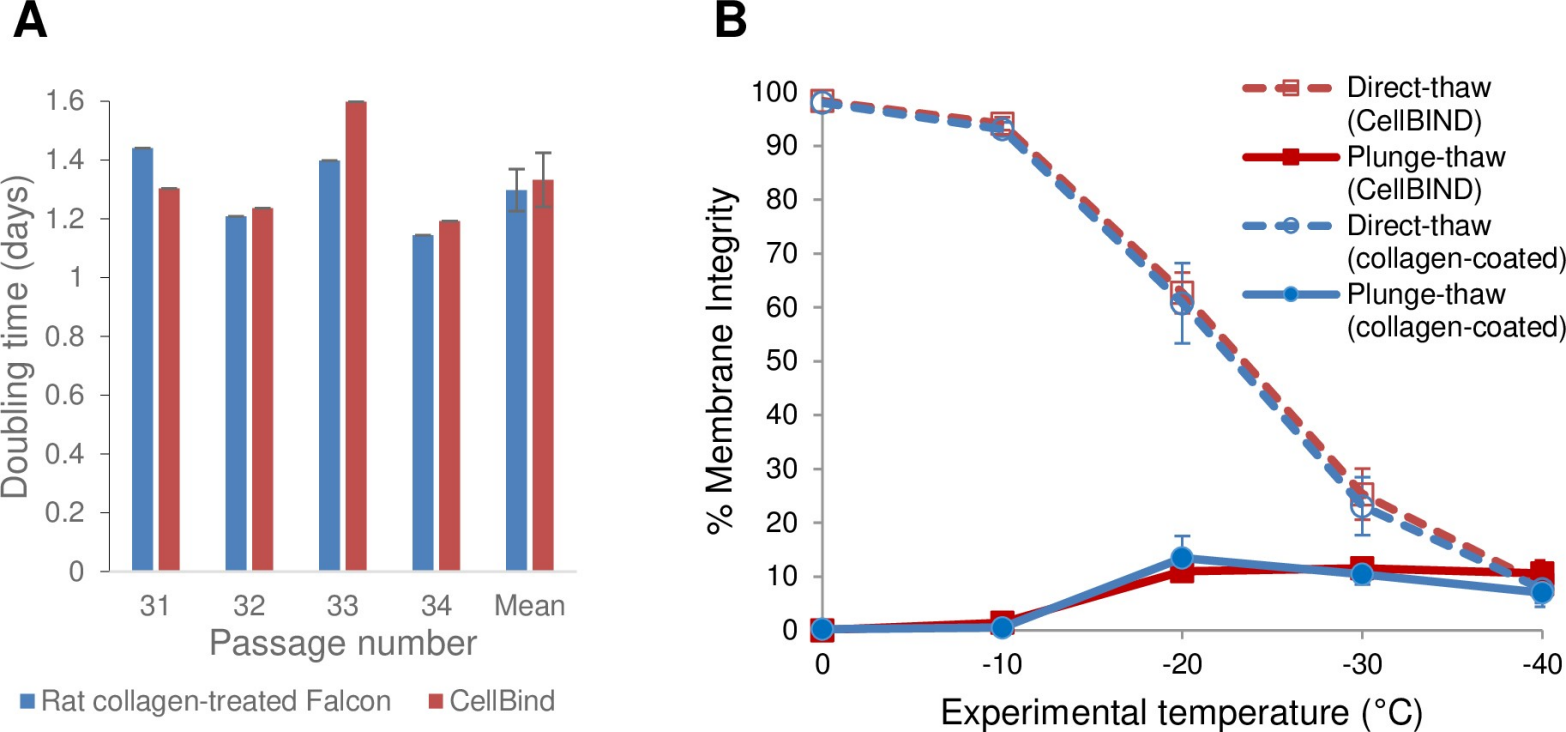
Growth kinetics and cryobiological response of hCMEC/D3 cells. (A) Doubling time (days) for hCMEC/D3 cells grown in rat collagen-treated Falcon flasks or Corning CellBIND flasks. (B) Graded freezing of hCMEC/D3 cells in suspension in the absence of cryoprotectants. The cells were cultured in either CellBIND flasks (red lines) or rat collagen-treated Falcon flasks (blue lines). Cooling was carried out at 1°C/min. At each experimental temperature, one sample was thawed directly (direct-thaw; dashed lines), and another sample was immediately plunged into liquid nitrogen, then thawed (plunge-thaw; solid lines). Membrane integrities (mean ± standard error of the mean (SEM) from three independent experiments) of direct-thaw and plunge-thaw samples at all experimental temperatures were not significantly different (p > 0.05) between cells grown in the two flasks.

The supplier of the hCMEC/D3 cell line recommends pre-coating cell culture vessels with rat collagen I. We found an alternative culture flask from Corning (CellBIND), whose plasma treatment creates a uniform surface with enhanced cell attachment and survival with no need for matrix protein coating [51]. The CellBIND surface has been shown to promote adhesion of transfected mammalian cell lines used for production of recombinant proteins [58]. Furthermore, a comparison of commercial culture plates with different growth surfaces revealed that CellBIND showed the lowest tendency of detachment of hepatocyte monolayers [59]. We compared the growth kinetics of hCMEC/D3 cultured on these two different flasks. Fig 1A shows that hCMEC/D3 cells grew equally well in the collagen-treated and CellBIND flasks (mean doubling time: 1.3 days). Statistical analysis for doubling times showed that they were not significantly different (p = 0.77). Because cell culture conditions are crucial to maintain reliability and reproducibility of experimental data it was important to establish that the cryobiological response of hCMEC/D3 cells was not affected by the vessel surface on which they were cultured. Therefore, we compared how hCMEC/D3 cells grown on rat collagen I-coated Falcon flasks *vs*. CellBIND flasks respond to freezing in the absence of cryoprotective agents. Fig 1B shows the post-thaw percent membrane integrity of hCMEC/D3 cells in suspension as they were slowly cooled at 1°C/min from 0°C to –40°C. Samples were either directly thawed (dashed red and blue lines for CellBIND flasks and rat collagen-treated flasks, respectively) or plunged into liquid nitrogen and then thawed (solid lines). The direct-thaw lines show that the percent membrane integrity was very high (> 90%) at the beginning of the cooling protocol when cells were at 0°C, but steadily declined with decreasing temperature. Progressive cell damage caused by extended exposure to increased solute concentration as temperature decreases and more ice forms extracellularly is manifested in the gradual decrease in membrane integrity. The plunge-thaw lines show that cells directly plunged into liquid nitrogen from 0°C experience rapid cooling injury. The membrane integrity of plunge-thaw samples remained low for all the temperatures tested but showed a slight increase at –20°C and lower temperatures. This suggests that with sufficient slow cooling cells are conferred some protection against injury during plunge into liquid nitrogen by losing intracellular water and reducing intracellular ice damage. Thus, hCMEC/D3 cells in suspension are sensitive to slow cooling injury based on the decline in membrane integrity of direct-thaw samples, as well as to rapid cooling injury, based on the decrease in membrane integrity upon plunge from temperatures between 0°C and −30°C when the cells have not been sufficiently slow cooled. More importantly, these results show that in the absence of CPAs there was no significant difference in % membrane integrity (p > 0.05) between direct-thaw samples (dashed lines) and between plunge-thaw samples (solid lines) whether collagen-coated Falcon flasks or CellBIND flasks were used to culture hCMEC/D3 cells. Therefore, in subsequent experiments, hCMEC/D3 cells were cultured in CellBIND flasks for convenience, efficiency, and quality-controlled surface treatment.

### Cryopreservation of hCMEC/D3 cells in suspension with CPAs

**Fig 2:**
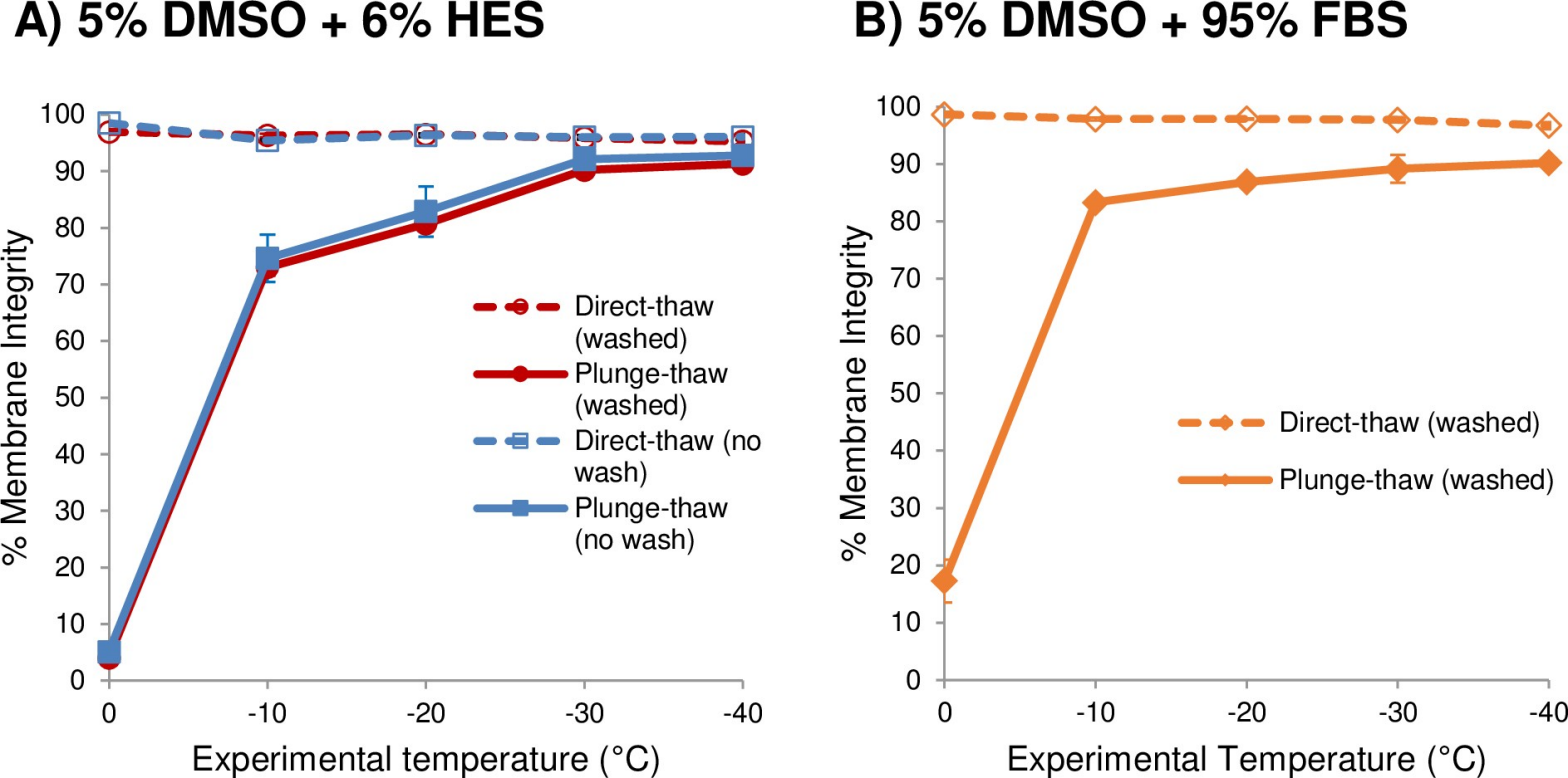
Graded freezing of hCMEC/D3 cells in suspension in the presence of CPAs. (A) 5% DMSO and 6% HES in medium or (B) 5% DMSO in 95% FBS. Cells were cooled at 1°C/min to sub-zero temperatures. At each temperature, one sample was thawed directly (direct-thaw, dashed lines), and another sample was immediately plunged into liquid nitrogen, and then thawed (plunge-thaw, solid lines). In (A) the cells were either washed or not washed after thaw. Washing was carried out by centrifugation at 140 g for 5 min, and removal of the supernatant before re-suspending the pellet in 400 μL PBS. Membrane integrities are shown as mean ± SEM from three independent experiments. Membrane integrity was not significantly different between cells that were washed or not washed post-thaw (p > 0.05). In (A), there was a significant difference between plunging from –10°C and –30°C or –40°C (p < 0.015 for no wash samples and p = 0.0004 for washed samples). In (B), there was no significant difference between plunging from –10°C and –30°C (p = 0.08), but there was a significant difference between plunging from –10°C and –40°C (p = 0.0012).

Previously, we showed an optimal protocol for the cryopreservation of endothelial cells (HUVECs, porcine and human corneal endothelial cells) in suspension. It involved loading the cells with cryopreservation solution containing 5% (w/w) DMSO and 6% (w/w) HES in cell medium at 0°C for 15 min, ice nucleation at –5°C, and cooling at –1°C/minute to a sufficiently low subzero temperature before plunge into liquid nitrogen [37, 38]. By applying this procedure [60] to hCMEC/D3 cells in suspension, we found that the same CPA combination sufficiently mitigated slow cooling injury as shown by the high % membrane integrity (~95%) of direct-thaw samples along the entire experimental temperature profile (Fig 2A, dashed lines). Furthermore, the CPA combination sufficiently diminished rapid cooling injury, as shown by the fact that after slow cooling to –40°C, no further loss of cell viability was incurred by plunging cells into liquid nitrogen and a membrane integrity of almost 93% was retained (Fig 2A, solid lines). This indicates that the cells have undergone adequate slow cooling dehydration to avoid intracellular ice formation injury.

The current cryopreservation protocol for hCMEC/D3 cells recommended by the supplier uses 5% DMSO in 95% FBS [50]. The supplier did not specify the rate of cooling and cryovial materials, but indicated that these cells were “placed at −80°C overnight and the next morning transferred in liquid nitrogen” [50]. We subjected the cells to the same graded freezing technique as before (as described in the legend for Fig 2A) except that we used 5% DMSO in 95% FBS as the CPA (Fig 2B). We obtained a maximum membrane integrity of 90.2 ± 0.6%, which was not significantly different (p = 0.09) from that which we obtained with 5% DMSO and 6% HES (92.8 ± 1.0%). Preliminary results showed that non-removal of FBS before the flow cytometric MI assessment resulted in a substantial amount of debris in the background fluorescence, which masked the green fluorescence of live cells. Therefore, it was necessary to wash the cells after thaw and before staining. In order to maintain consistency, we also washed the cells cryopreserved with 5% DMSO and 6% HES, but as shown in Fig 2A, washing did not affect the membrane integrity.

Although membrane integrity is viewed as a reliable marker of cell viability after cryopreservation [61], we also carried out functional assessments post-thaw and compared the results with those for fresh unfrozen cells. Some cells were sub-cultured to confluence, and then immunohistochemistry was carried out to detect the tight junction protein ZO-1, while other cells were seeded on Matrigel for a tube formation assay.

**Fig 3:**
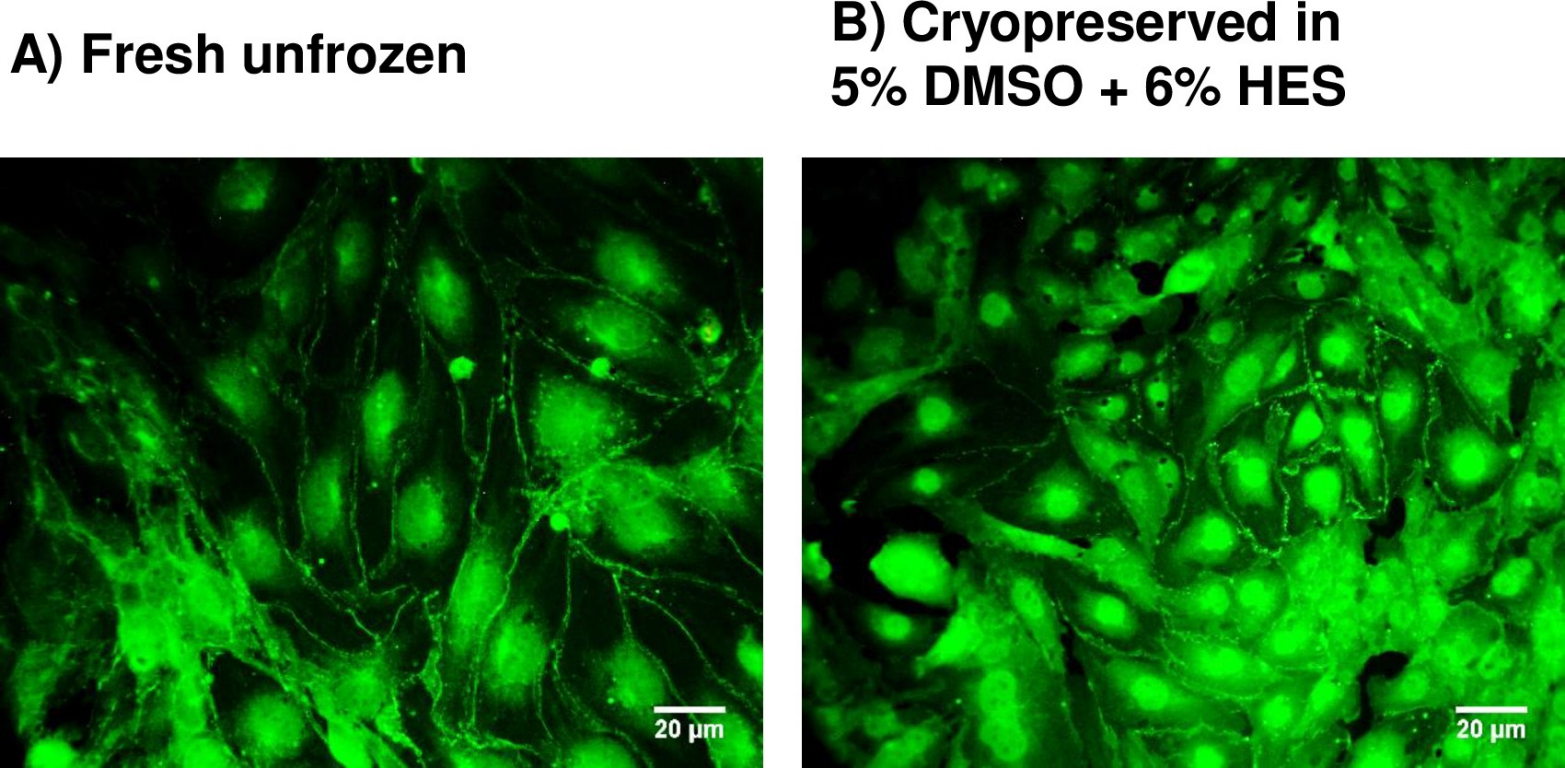
Expression of ZO-1 in hCMEC/D3 cells. (A) Non-cryopreserved (fresh, unfrozen), or (B) cryopreserved in suspension by slow cooling in the presence of 5% DMSO and 6% HES, and then plunging into liquid nitrogen, and then rapidly thawed. Fresh cells and cryopreserved cells in suspension were seeded onto fibronectin-coated glass coverslips and allowed to reach confluency. The cell monolayers stained with ZO-1 antibody conjugated to Alexa Fluor 488 were imaged by fluorescent microscopy.

Fig 3 shows a comparison in ZO-1 expression between fresh unfrozen and post-thawed hCMEC/D3 cells in suspension that were subsequently grown into monolayers. The continuous expression of ZO-1 along the periphery of adjoining cells in both unfrozen *vs*. cryopreserved hCMEC/D3 cells indicates that the ability to express this tight junction protein was retained after cryopreservation.

**Fig 4:**
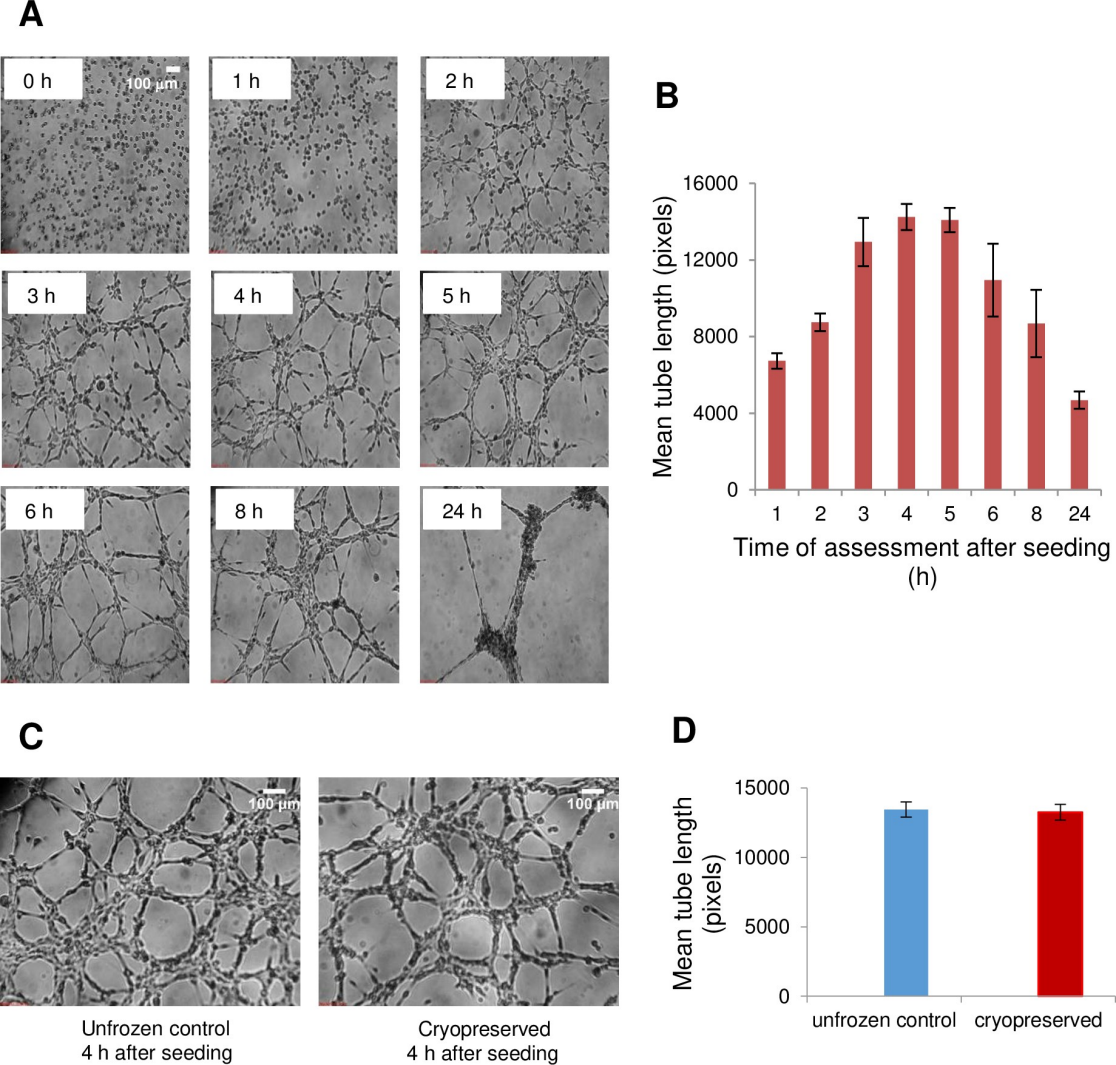
Tube formation of hCMEC/D3 cells in suspension seeded on Matrigel. (A) Representative phase-contrast images (40X magnification) of tube formation by unfrozen cells as a function of time (hours after seeding). The scale bar at 0 h applies to all time points. (B) Quantification of mean total tube length. The error bars indicate the SEM from six to 12 images analyzed per time point. (C) Comparison of tube-forming ability of unfrozen or cryopreserved hCMEC/D3 cells in suspension 4 h after seeding. Cryopreserved cells were loaded with 5% DMSO and 6% HES, cooled at 1°C/min to –40°C, plunged into liquid nitrogen, and rapidly thawed. Equal numbers (15,000 cells per well) of unfrozen and cryopreserved cells were plated on Matrigel. (D) Mean tube lengths; error bars indicate the SEM from 18 images each from independent experiments (n = 4 for unfrozen control cells, n = 3 for cryopreserved cells).

We also used the *in vitro* tube formation assay to quantitate the angiogenic ability of hCMEC/D3 cells to form capillary-like tubular structures when cultured on the reconstituted basement membrane [61]. Fig 4A shows the time-dependent tube formation of hCMEC/D3 cells. Fig 4B shows that tube length is a function of time, and that the maximum tube formation was attained 4 hours after seeding. We then assessed tube formation of fresh *vs*. cryopreserved hCMEC/D3 cells in suspension 4 h after seeding (Fig 4C) and found that there was no significant difference between them (p = 0.8) (Fig 4D). Therefore, hCMEC/D3 cells in suspension retained their angiogenic function after cryopreservation in 5% DMSO and 6% HES.

### Cryopreservation of astrocytes in suspension with CPAs

**Fig 5:**
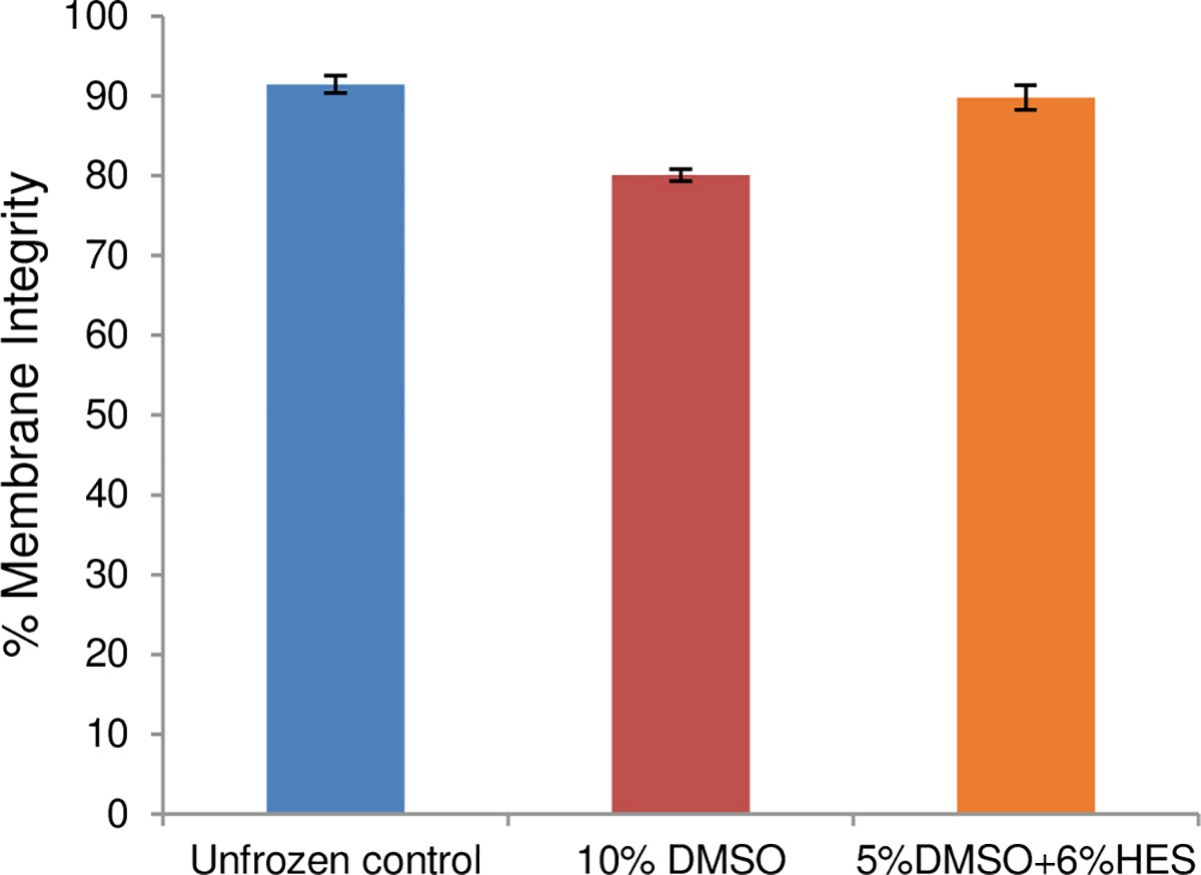
Cryopreservation of astrocytes in suspension. Comparison of membrane integrity of unfrozen astrocytes in suspension and those cooled at 1°C/min to –40°C in the presence of 10% DMSO *vs*. 5% DMSO and 6% HES, plunged into liquid nitrogen, and rapidly thawed. Data are shown as mean ± SEM from two independent experiments using four samples for each condition.

Next, we applied the same slow cooling procedure [60] to astrocytes in suspension and compared the membrane integrity of unfrozen control cells to those cryopreserved in the presence of 10% DMSO (as used by the manufacturer [62]) *vs*. 5% DMSO + 6% HES (our optimized CPA combination for endothelial cells [37, 38, 60]). The astrocytes were loaded for 15 min at 0°C with a cryopreservation solution (containing either 10% (w/w) DMSO or 5% (w/w) DMSO and 6% (w/w) HES in cell medium); ice was nucleated at –5°C, and cells were cooled at –1°C/min to –40°C, plunged into and stored in liquid nitrogen, and rapidly thawed. Fig 5 shows that a significantly higher (p = 0.001) post-thaw membrane integrity was attained using 5% DMSO + 6% HES (89.8 ± 1.5%) compared to using 10% DMSO (80.1 ± 0.7%). Moreover, astrocytes in suspension cryopreserved in 5% DMSO + 6% HES had post-thaw membrane integrity that is not statistically different (p = 0.41) from that of fresh unfrozen control (91.5 ± 1.1%).

### Cryopreservation of hCMEC/D3 cells in monolayers

Previously, we developed a breakthrough procedure for cryopreservation of monolayers of HUVECs or porcine corneal endothelial cells [46, 48]. We showed that high membrane integrity can be obtained by culturing these cells on fibronectin-coated Rinzl plastic coverslips and slowly cooling (at 0.2°C/min or 1°C/min) in the presence of 5% DMSO, 6% HES, and 2% chondroitin sulfate (CS) before storage in liquid nitrogen [46, 48]. Here we seeded hCMEC/D3 cells onto fibronectin-coated coverslips made of three different substrates namely Rinzl, glass, and Thermanox. The cells were allowed to reach confluence, and then the coverslips with cell monolayers were transferred to glass vials. The CPA combination (5% DMSO + 6% HES + 2% CS in medium) was loaded to the cell monolayers for 15 min at 0°C and then ice nucleation was induced at –5°C. Controlled slow cooling (–1°C/min) was carried out until –40°C, after which the glass vials were transferred to liquid nitrogen for storage. Rapid thaw was performed in a 37°C water bath, and the CPA was removed using a single wash with 1XPBS. Because the effect of cryopreservation on cell viability and function does not always manifest itself immediately after thaw, it was necessary to determine whether the hCMEC/D3 cell monolayers retain their viability and metabolic activity after extended post-thaw incubation. One set of cell monolayers was analyzed for membrane integrity by SYTO 13/GelRed staining immediately post-thaw (same day), while another set was incubated post-thaw at 37°C overnight then analyzed (next day). SYTO 13/GelRed-labeled nuclei of membrane-intact cells fluoresce green and membrane-damaged cells fluoresce red. Yellowish-colored cells indicate double staining and were counted as membrane-damaged. Cell membrane integrity (also referred to as relative viability) and absolute viability in each captured image were calculated as defined in Eqs 3 and 4 above. Similarly, one set of monolayers was analyzed by AlamarBlue reduction assay the same day and after overnight incubation. The AlamarBlue color change provides a quantitative measure of the mitochondrial activity of the cells.

**Fig 6:**
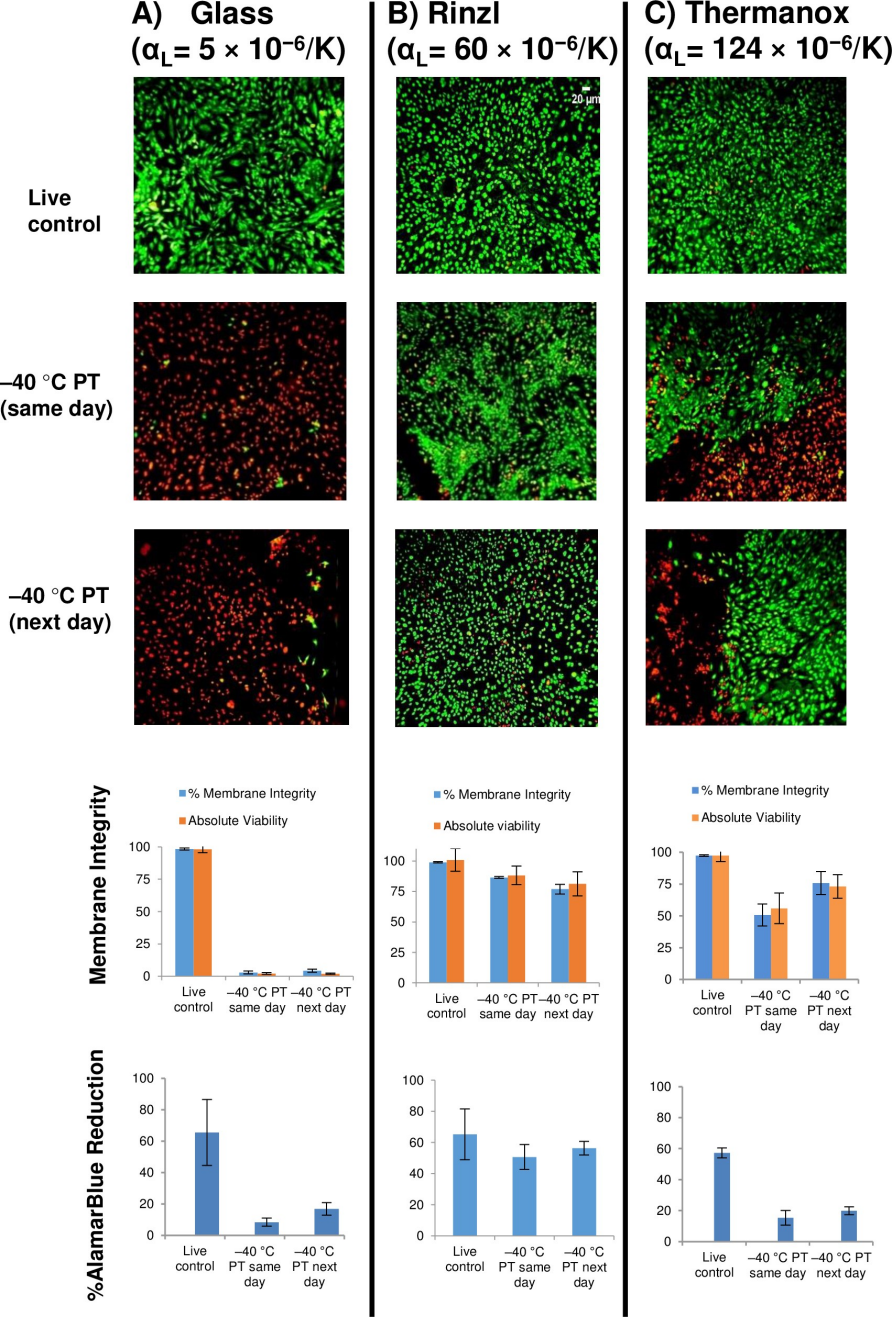
Cryopreservation of hCMEC/D3 monolayers on (A) glass, (B) Rinzl, and (C) Thermanox coverslips. Live control samples were unfrozen and the frozen samples (labelled –40°C PT) were cooled at 1°C/min to –40°C in the presence of 5% DMSO, 6% HES, and 2% chondroitin sulfate, plunged and stored in liquid nitrogen, rapidly thawed, and assessed either on the same day or after overnight incubation (next day). Shown are fluorescent microscope images of hCMEC/D3 monolayers stained with SYTO 13 and GelRed (scale bar for live control cells on Rinzl applies to all images), percent membrane integrities (blue bars) and absolute viabilities (orange bars), and metabolic activity as assessed by AlamarBlue reduction assay. Data represent the mean of three independent experiments ± SEM.

Fig 6 depicts representative SYTO 13/GelRed fluorescent images, membrane integrity, and metabolic activity (AlamarBlue reduction) of hCMEC/D3 cells cultured on coverslips made of glass (Fig 6A, left column), Rinzl (Fig 6B, middle column) or Thermanox (Fig 6C, right column). The coefficient of linear thermal expansion (α_L_) of each underlying solid substrate is indicated. The fluorescent images show that in the presence of CPA (5% DMSO + 6% HES + 2% CS), fresh unfrozen cell monolayers (live control) seeded on all three substrates are mainly membrane-intact (SYTO 13-positive, green) indicating that the CPA combination did not pose any toxic effect. Cell monolayers that were immediately plunged into liquid nitrogen in the absence of CPA are predominantly membrane-damaged (GelRed-positive) regardless of the underlying solid substrate (not shown).

We compared the response to slow cooling of hCMEC/D3 monolayers cultured on the different substrates. Post thaw, monolayers on Rinzl with the optimal coefficient of linear thermal expansion matched to ice (Fig 6B, middle column) have mainly green cells, a high percent membrane integrity (86.5 ± 0.7%) and absolute viability (88.2 ± 7.5%) on the same day of assay that were not statistically different (p = 0.08 and 0.6, respectively) after overnight incubation (76.9 ± 4% membrane integrity and 81.3 ± 9.8% absolute viability). Moreover, the metabolic activity of the unfrozen control (65.3 ± 16.3% AlamarBlue reduction) was not statistically different (p = 0.5) from that of the frozen hCMEC/D3 monolayers that were assessed the same day (50.6 ± 8% AlamarBlue reduction), or incubated overnight post-thaw (56.3 ± 4.4% AlamarBlue reduction; p = 0.6). On the other hand, hCMEC/D3 cell monolayers on fibronectin-coated glass coverslips with a lower than optimal coefficient of linear thermal expansion (Fig 6A, left column) did not survive cryopreservation even in the presence of CPA, as shown by the mostly red cells, minimal viability and metabolic activity, on both the same day and next day post-thaw assessments. Similarly, only some of the hCMEC/D3 cells cultured on Thermanox with a higher than optimal coefficient of linear thermal expansion (Fig 6C, right column) remained viable post-thaw on the same day of assay as well as after post-thaw overnight incubation at 37°C (next day). There was a reduction in both percent membrane integrity and absolute viability post-thaw compared to the live control for same day assay. There appears to be a slight recovery in cell viability after overnight incubation. Metabolic activity by AlamarBlue reduction assay was significantly reduced (p = 0.009) in cryopreserved monolayers relative to the unfrozen control, although there was a slight increase after overnight incubation consistent with the observed recovery in cell viability. Because, of the three substrates, Rinzl showed the best post-thaw outcome, cells were cultured on Rinzl coverslips for the rest of the monolayer experiments.

**Fig 7:**
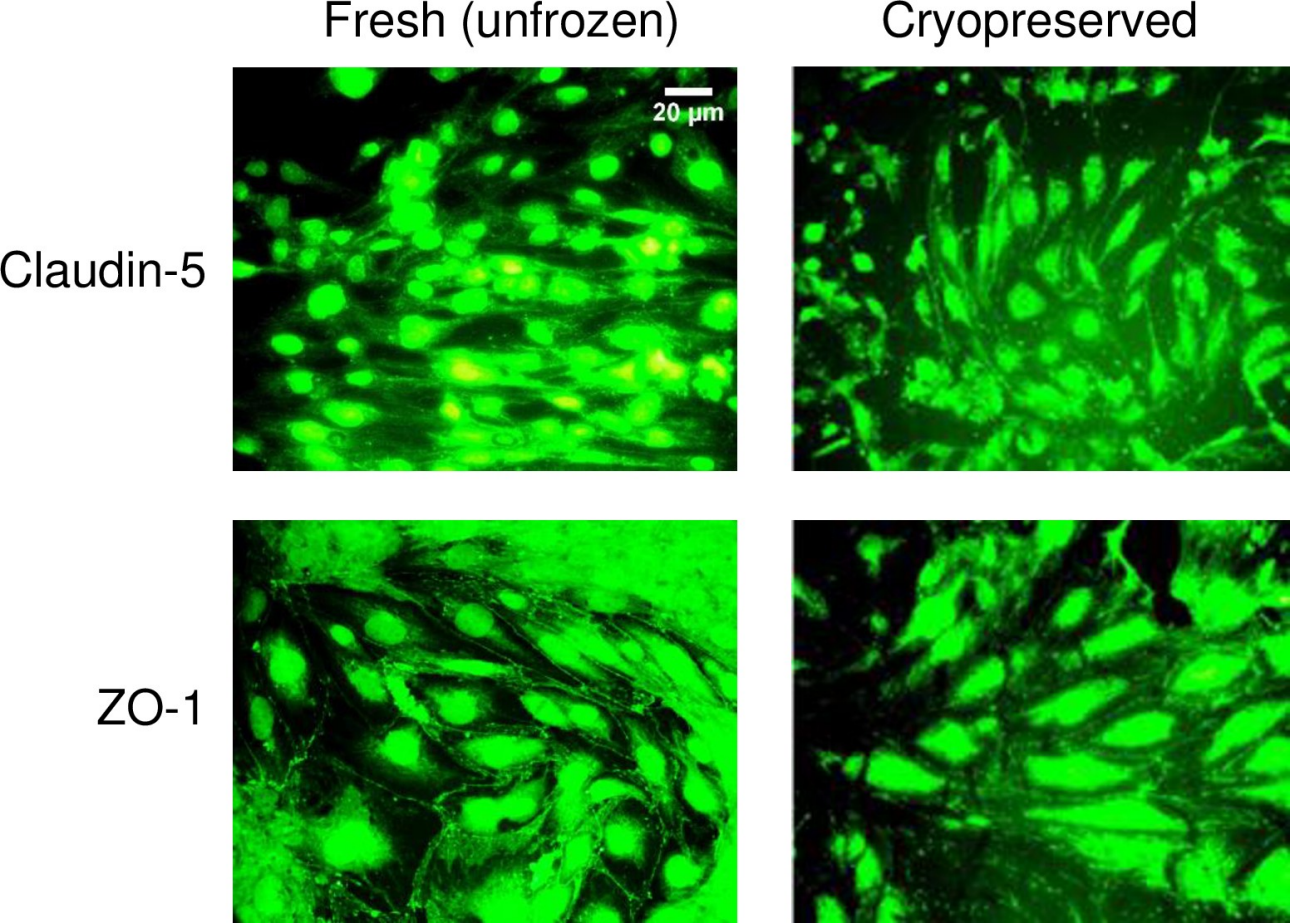
Junction protein expression in fresh and cryopreserved hCMEC/D3 monolayers. The expression of claudin-5 and zonula occludens (ZO)-1 in hCMEC/D3 monolayers that were non-cryopreserved (fresh, unfrozen control), or were loaded with 5% DMSO, 6% HES, and 2% chondroitin sulfate, subjected to cooling at 1°C/min to –40°C, plunging and storage in liquid nitrogen, rapid thawing, and immediate cryoprotectant removal. Fresh and cryopreserved monolayers were stained with the corresponding antibody conjugated to Alexa Fluor 488. The scale bar for claudin-5-stained fresh, unfrozen cells applies to all images.

Aside from viability and metabolic function, we also examined structural markers of hCMEC/D3 monolayers before and after cryopreservation. Immunocytochemical staining revealed the presence of the tight junction proteins claudin-5 and ZO-1 along the borders of adjacent cells in both fresh and cryopreserved monolayers indicating the retention of these markers after cryopreservation (Fig 7).

### Cryopreservation of astrocytes in monolayers

**Fig 8:**
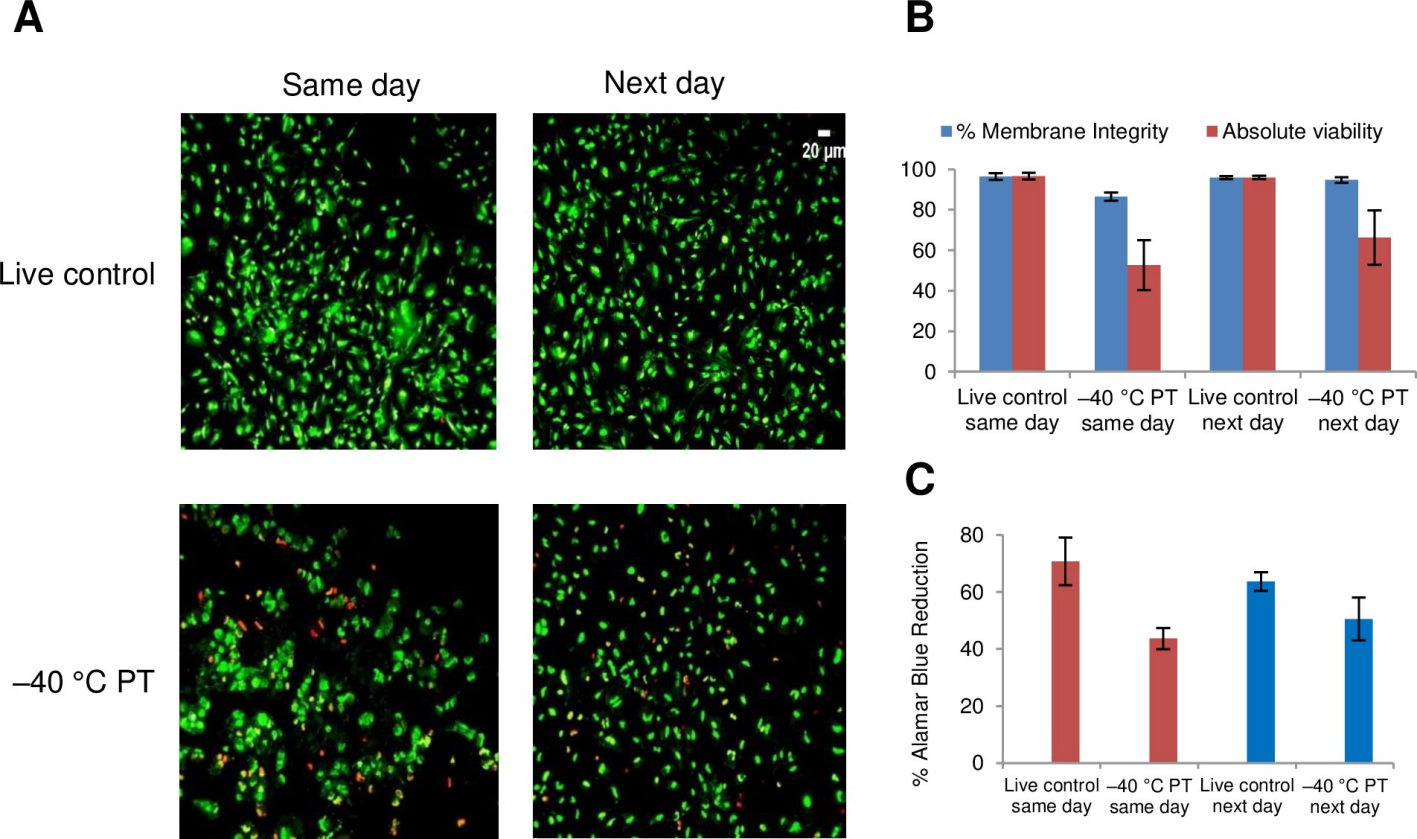
Cryopreservation of astrocyte monolayers. (A) Fluorescent microscope images of astrocyte monolayers on fibronectin-coated Rinzl coverslips. Live control samples were unfrozen, and the frozen samples (labelled –40°C PT) were cooled at 1°C/min to –40°C in the presence of 5% DMSO, 6% HES, and 2% chondroitin sulfate, plunged and stored in liquid nitrogen, and rapidly thawed. Cryoprotectant was removed with a single PBS wash, and the monolayers assessed either on the same day of thaw or after post-thaw overnight incubation (next day). The monolayers were stained with SYTO 13 and GelRed; scale bar for live control, next day, applies to all images. (B) Percent membrane integrity (blue bars) and absolute viability (red bars) in each captured image were quantified using Viability3 automated cell counting software. Data represent the mean ± SEM of three independent experiments using three to five coverslips per condition. (C) Metabolic activity of astrocyte monolayers by AlamarBlue reduction. Data represent the mean ± SEM of three independent experiments using four coverslips per condition.

Next, we investigated the cryobiological response of astrocytes in monolayers. We seeded primary human astrocytes onto fibronectin-covered Rinzl coverslips, allowed the cells to reach confluence, and then transferred the cell monolayers to glass vials. CPA (5% DMSO + 6% HES + 2% CS) was loaded to the cell monolayers for 15 min at 0°C and then ice nucleation was induced at –5°C. Controlled slow cooling (–1°C/min) was carried out until –40°C, after which the glass vials were transferred to liquid nitrogen for storage. Rapid thaw was performed in a 37°C water bath, and the CPA was removed using a single wash with 1XPBS. The cell monolayers were immediately stained with SYTO13/GelRed and imaged for membrane integrity immediately post-thaw or after post-thaw overnight incubation in medium at 37°C as previously described [46]. Fig 8A shows representative images of SYTO 13/GelRed stained astrocyte monolayers with or without (live control) undergoing cryopreservation. Cell monolayers that were unfrozen (live control) are predominantly green (viable) both on the same day post-thaw and after post-thaw overnight incubation (next day). Astrocyte monolayers slowly cooled before plunge in liquid nitrogen showed areas where cells have detached or whose cell membranes have been damaged. Fig 8B confirms that post-thaw membrane integrity remained high and similar to the viability of live controls, consistent with cell nuclei mostly stained green (live) both on same day (86.5 ± 2.1%) and after overnight incubation (94.7 ± 1.3%) at 37°C (next day). However, absolute viabilities were reduced after cryopreservation (52.6 ± 12.3% as assessed on the same day, and 66.3 ± 13.5% the next day, p = 0.5). The lower absolute viabilities were consistent with the observed cell detachment shown in the images in Fig 8A. Fig 8C shows that metabolic activity by AlamarBlue reduction was decreased significantly following cryopreservation of monolayers assayed on the same day (p = 0.02). Interestingly, after post-thaw overnight incubation, the cryopreserved astrocyte monolayers appear to recover their metabolic activity and were able to reduce AlamarBlue to the extent not significantly different from their live control counterparts (p = 0.16).

### Cryopreservation of hCMEC/D3 and astrocyte monolayer co-cultures

**Fig 9:**
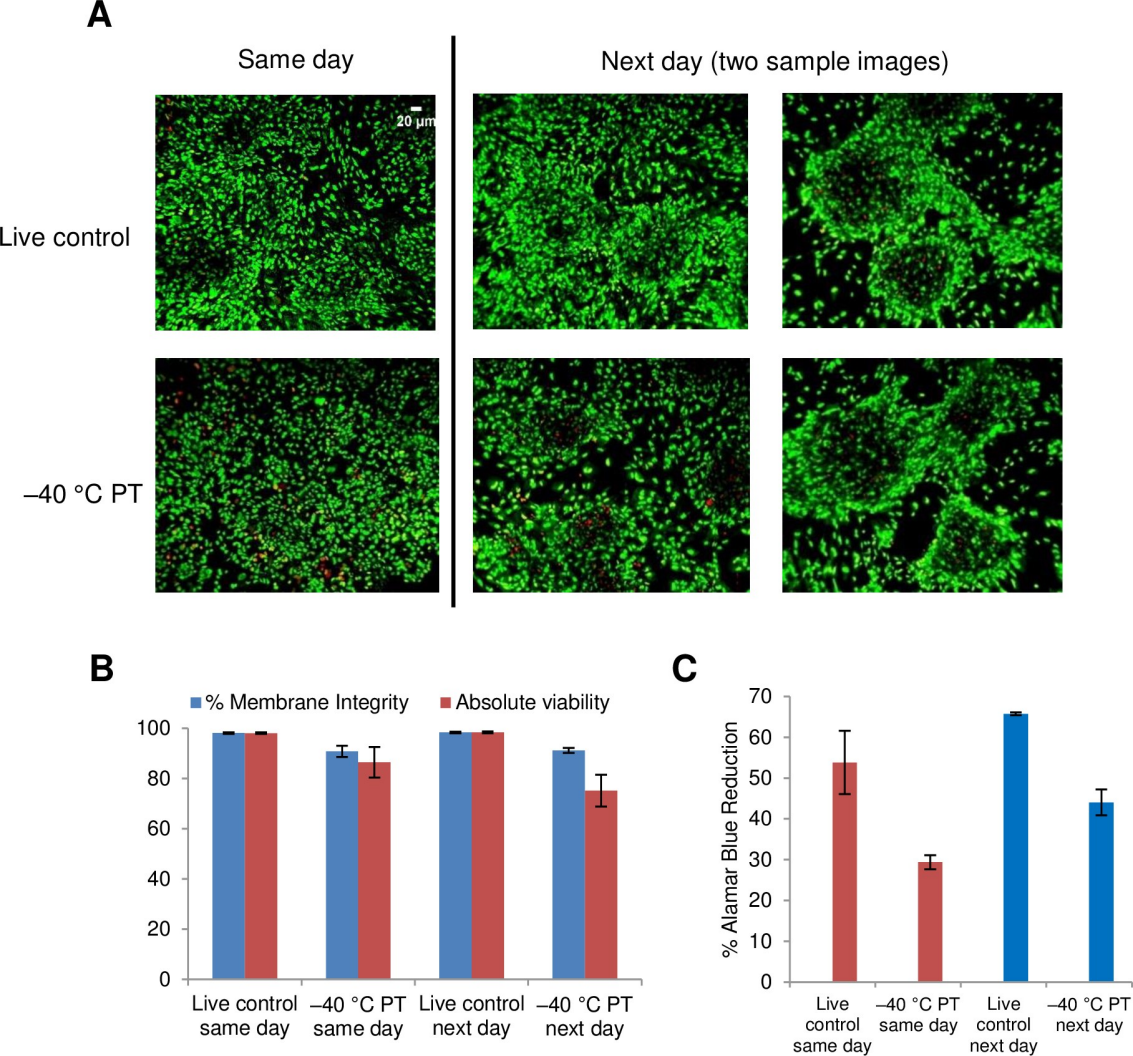
Cryopreservation of hCMEC/D3 and astrocyte co-cultures. (A) Fluorescent microscope images of hCMEC/D3 and astrocyte co-cultures on fibronectin-coated Rinzl coverslips. Live control samples were unfrozen, and the frozen samples (labelled –40°C PT) were cooled at 1°C/min to –40°C in the presence of 5% DMSO, 6% HES, and 2% chondroitin sulfate, plunged and stored in liquid nitrogen, and rapidly thawed. Cryoprotectant was removed with a single PBS wash, and the monolayers assessed either on the same day or after post-thaw overnight incubation (next day). The monolayers were stained with SYTO 13 and GelRed; scale bar for live control, same day, applies to all images. Next day images show spheroid formation. (B) Percent membrane integrity (blue bars) and absolute viability (red bars) in each captured image were quantified using Viability3 automated cell counting software. Data represent the mean ± SEM of two independent experiments using three to four coverslips per condition. (C) Metabolic activity of hCMEC/D3 and astrocyte co-cultures. Data represent the mean ± SEM of two independent experiments using three to four coverslips per condition.

Next, we co-cultured equal numbers of hCMEC/D3 cells and astrocytes to confluence on fibronectin-coated Rinzl coverslips. The monolayers were either fresh unfrozen (live control) or cryopreserved (loaded for 15 min at 0°C with 5% DMSO + 6% HES + 2% CS, ice nucleated at –5°C, cooled at –1°C/min to –40°C before plunge into, and storage in liquid nitrogen) and rapidly thawed. They were assayed on the same day (Fig 9A, left two panels) or after post-thaw overnight incubation at 37°C (Fig 9A, right four panels). The fresh co-culture monolayers stained with SYTO 13/GelRed were predominantly membrane-intact (green), while a few membrane-damaged (red) cells were evident after cryopreservation, as assayed both on the same day and the next day. Fig 9B shows that the membrane integrity of cryopreserved monolayers remained high (> 90%) compared to the unfrozen controls when assessed immediately post-thaw on the same day and the next day. However, the membrane integrity was significantly reduced after cryopreservation as assessed on the same day (p = 0.024) as well as the next day (p = 0.0005). Fig 9C shows that the metabolic activity by AlamarBlue reduction decreased significantly following cryopreservation as assessed on the same day (p = 0.02) and the next day (p = 0.001), but showed significant (p = 0.0065) recovery after overnight incubation post-thaw. Interestingly, the hCMEC/D3 cells and astrocytes appear to self-assemble into spheroid-like structures following overnight incubation, with a few dead cells detected inside the spheroid, and this behavior was retained after cryopreservation (Fig 9A, right panels).

## Discussion

Cryopreservation of human cerebral microvascular endothelial cells and astrocytes is important for their availability for research on the blood–brain barrier. Although they are currently obtainable commercially as frozen cells in suspension, a better understanding and mitigation of cryoinjury can improve their post-thaw viability and enhance their distribution and accessibility. Moreover, because these cells exist *in vivo* as confluent monolayers, a practical protocol of cryopreservation in this two-dimensional configuration will allow their availability as a ready-to-use experimental platform. In addition, it may be possible to deliver on-demand brain microvascular endothelial cells and astrocytes as cryopreserved monolayer co-cultures that maintain their native association with each other in the blood–brain barrier.

### Cryopreservation of hCMEC/D3 cells in suspension

Our first set of experiments focused on cryopreservation of hCMEC/D3 cells in suspension, i.e., dispersed in medium. By using graded freezing or interrupted slow cooling [35–38, 52, 60, 63], we show that hCMEC/D3 cells in suspension are sensitive to injury from solute effects based on the decline in membrane integrity as cells were slowly cooled (Fig 1B). In addition, hCMEC/D3 cells in suspension retrieved at various temperatures along the cooling profile and immediately plunged into liquid nitrogen experience rapid cooling injury due to intracellular ice formation. The observation that about 10% of cells retained their membrane integrity at lower subzero temperatures (≤ –20°C) suggests that some intracellular water was released by the cells during the slow cooling prior to the liquid nitrogen plunge. Our results indicate that hCMEC/D3 cells in suspension incurred both slow-cooling and rapid cooling injury [23] and suggest the addition of a penetrating CPA and a non-penetrating CPA, respectively in order to mitigate these injuries. We used a combination of 5% DMSO and 6% HES in media as we had optimized for other endothelial cell types in suspension [37, 38], and show that hCMEC/D3 cells in suspension cooled at 1°C/min to –40°C before plunge into liquid nitrogen had a post-thaw membrane integrity of 92.8 ± 1.0% (Fig 2A). The cell supplier recommends freezing hCMEC/D3 cells in the presence of 5% DMSO and 95% FBS [50]. Therefore, we also cooled hCMEC/D3 cells in suspension in the presence of 5% DMSO and 95% FBS at 1°C/min to –40°C before plunge in liquid nitrogen, and we obtained a post-thaw membrane integrity of 90.2 ± 0.6% (Fig 2B). Given the high membrane integrity values for both protocols, further optimization was deemed unnecessary. FBS is a routine media supplement that provides a complex range of proteins and growth factors for cells to proliferate in culture [64]. It can act as a non-penetrating CPA due to the presence of large molecular components such as bovine serum albumin. However, because it is animal-derived, source- and batch-variable, expensive, and non-chemically defined, it is falling out of favor from current good lab practices especially for clinical applications. On the other hand, HES offers the advantages of being chemically defined and having been approved for clinical use as a plasma volume expander [32]. Therefore, we believe that our CPA combination of 5% DMSO + 6% HES may be more desirable in some circumstances than 5% DMSO + 95% FBS for cryopreserving hCMEC/D3 cells in suspension.

It is important to note that the cryopreservation procedure was performed using borosilicate glass tubes that are not leak-proof for long-term storage in liquid nitrogen. Aside from differences in volumes of cell suspension used (glass tubes contained 0.2 mL while plastic cryovials can contain 1.0 mL), the thermal conductivity of plastic is lower than glass. In a previous work [52], we modified the freezing procedure when using plastic cryovials, i.e., ice nucleation was induced at –8°C and the latent heat of fusion was allowed to be released for 5 min. We showed no statistically significant difference in the post-thaw viability of cells that were cryopreserved in glass tubes *vs*. plastic cryovials.

Although membrane integrity is an acceptable indicator of cell viability, we also evaluated specific functions that brain microvascular cells perform *in vivo*. Our previous studies show that membrane integrity values over 60% are associated with positive measures of cell functionality [61]. First, we show that both unfrozen and cryopreserved hCMEC/D3 cells in suspension grown into monolayers express the cell junction protein ZO-1 (Fig 3). This tight junction protein is important in the function of the blood–brain barrier in maintaining homeostasis by restricting non-specific flux of ions, molecules, and cells into and out of the central nervous system [65]. Another characteristic *in vivo* function of vascular endothelial cells is angiogenesis, the formation of new blood vessels from existing ones, a process which can be recapitulated *in vitro* using the Matrigel tube formation assay [39]. Here, we show that hCMEC/D3 cells cryopreserved in suspension showed an ability to form tubes on Matrigel similar to the unfrozen control (Fig 4C). This finding may have important implications because a disruption of the BBB is one of the pathophysiological features of ischemic stroke [5, 66], and induction of angiogenesis is an emerging strategy for recovery [67]. In fact, it has been shown in a mouse cerebral ischemia model that angiogenesis occurred immediately after injury, and 42 angiogenesis genes and several angiogenic growth factors were increased within an hour [68]. Indeed, we demonstrate here that tubes started to form within an hour of seeding hCMEC/D3 on Matrigel, reaching a peak at 4 h (Fig 4A and 4B). Therefore, the results of our immunohistochemical detection of ZO-1 and tube formation assay show that hCMEC/D3 cells were functional following their cryopreservation in suspension.

### Cryopreservation of astrocytes in suspension

Next, we cryopreserved astrocytes in suspension. We found that the combination of 5% DMSO + 6% HES is superior over 10% DMSO (CPA used by the supplier [62]) for maintaining cell viability after cryopreservation (Fig 5). The post-thaw membrane integrity obtained using 5% DMSO and 6% HES was 89.8 ± 3.1%, which was significantly higher than 80.1 ± 1.5% when 10% DMSO was used. The use of 10% DMSO is routine for many cell lines and primary cells; however, studies have demonstrated toxicity of DMSO through induction of programmed cell death [69, 70]. In fact, DMSO concentrations as low as 4% have been shown to inhibit mitochondrial respiration and induce early stages of apoptosis in a retinal neuronal cell line [71]. Moreover, the damaging effects of DMSO on the developing central nervous system have been shown in post-natal mice [72]. In particular, 12 h exposure of primary rat astrocytes to 5% and 10% DMSO were shown to eliminate astrocyte processes, and significantly reduce marker expression, cell number, and viability [73]. Therefore, we believe that our CPA combination of 5% DMSO + 6% HES is better than 10% DMSO for cryopreserving astrocytes in suspension.

### Cryopreservation of hCMEC/D3 cells and astrocytes in monolayers

Next, we cryopreserved hCMEC/D3 cells and astrocytes in monolayer configuration. Several factors add to the complexity of the cryopreservation of attached cells. Successful cryopreservation of cell monolayers depends on cell attachments (to other cells and to the underlying solid substrate) which can in turn increase intracellular ice formation [41]. Intracellular ice has been shown to spread from cell to cell in monolayers faster than cells in suspension can form intracellular ice [43, 74]. This has been attributed to gap junctions through which intracellular ice propagates [43]. It has also been shown that ice can grow into paracellular spaces between cells with tight junctions [75]. In addition, during cryopreservation and thawing, both ice and substrate experience contraction and expansion processes. In our previous work we suggested that because of the greater mismatch in the coefficient of linear thermal expansion (*α_L_*) between ice (51 × 10^−6^/K) and glass (5 × 10^−6^/K) compared to between ice and Rinzl (60 × 10^−6^/K), the compressive strain on cell monolayers on glass could lead to their detachment upon cooling and warming in the presence of ice [45]. In consideration of all these factors, we employed graded freezing to develop an optimized protocol for the cryopreservation of monolayers of HUVECs and porcine corneal endothelial cells [46, 48]. Applying the protocol here, hCMEC/D3 cells were grown to confluence on Rinzl, a substrate with a similar coefficient of thermal expansion to ice, which was coated with fibronectin for better attachment. The hCMEC/D3 monolayers were loaded with 5% DMSO and 6% HES supplemented with 2% chondroitin sulfate, ice-nucleated at −5°C, cooled at 1°C/min to –40°C, plunged into and stored in liquid nitrogen, rapidly thawed in a 37°C water bath, and the CPA removed by a single wash with PBS. The membrane integrity and absolute viability were maintained post thaw on the same day of experiment and after overnight incubation post-thaw of the hCMEC/D3 monolayers cultured on Rinzl (Fig 6B). As to be expected, cell monolayers on glass (with a lower coefficient of linear thermal expansion than that of ice) had very poor viability and metabolic activity after cryopreservation (Fig 6A) likely due to the differential contraction and expansion of ice and glass over the course of cooling and thawing [45]. We then examined whether replacing Rinzl, a rigid polyvinylchloride plastic characterized by high transparency, index of refraction, and toughness [76], with another similar substrate but with a higher coefficient of thermal expansion than that of ice will lead to the expected decline in post-thaw viability and function of monolayers. Thermanox, a flexible transparent plastic that has been tissue-culture treated for improved cell attachment and spreading [77], is described as a proprietary polyester whose coefficient of linear thermal expansion is higher than that of ice [47]. Here we show that compared to Rinzl, Thermanox is not a good substrate for cryopreserving monolayers (Fig 6B and 6C). It is possible that the greater difference in the *α_L_* values between Thermanox and ice *vs*. between Rinzl and ice created a greater mismatch strain on the monolayer that resulted in the reduction in cell viability and decreased metabolic activity that we observed here.

The metabolic ability, assessed by the ability to reduce AlamarBlue, of the frozen hCMEC/D3 monolayers on Rinzl incubated overnight post-thaw remained comparable to the unfrozen control and to those assessed immediately (same day). Furthermore, we also showed that ZO-1 expression was maintained in unfrozen and cryopreserved hCMEC/D3 monolayers. In addition, the expression of claudin-5, the most enriched tight junction protein in the blood–brain barrier [78], was retained after cryopreservation of hCMEC/D3 monolayers on Rinzl (Fig 7).

Astrocytes are the closest neighboring cells to the brain microvascular endothelial cells and their role in the blood–brain barrier and in neurodegenerative disorders remains an area of active investigation [79]. An *in vitro* model of astrocytes in monolayer configuration would be invaluable in these studies. We used human primary astrocytes in this work because they are expected to show the best functional performance; however, these cells are known to have poor proliferative potential and are prone to degeneration. Cryopreservation of astrocyte monolayers would overcome their limited availability and avoid unwanted differentiation during extensive passaging of cells in suspension. We employed the same protocol as in the cryopreservation of endothelial monolayers [46, 48]. Astrocytes were grown on fibronectin-coated Rinzl coverslips. The monolayers were loaded with 5% DMSO, 6% HES, and 2% chondroitin sulfate, ice-nucleated at −5°C, cooled at 1°C/min to –40°C, plunged into and stored in liquid nitrogen, rapidly thawed in a 37°C water bath, and the CPA removed by a single wash with PBS. It is noteworthy that the SYTO 13/GelRed-labeled nuclei of cells in the astrocyte monolayers were more dispersed compared to the nuclei of cells in hCMEC/D3 monolayers, because tight junctions do not connect astrocytes. Importantly, the membrane integrity of post-thaw monolayers was maintained compared to the unfrozen controls. However, cryopreservation decreased absolute viabilities due to some cell detachment, but the post-thaw monolayers retained metabolic activity after overnight incubation in cell medium.

In order to develop a more physiologically relevant cell culture model that recapitulates the intercellular interactions in the blood-brain barrier, we co-cultured hCMEC/D3 cells with astrocytes in a 1:1 ratio. Previously, it has been shown that co-cultures of brain endothelial cells with astrocytes enhanced the frequency and complexity of tight junctions which closely resembled their *in vivo* counterparts [80]. Here we showed that in the presence of 5% DMSO + 6% HES + 2% chondroitin sulfate cooling co-cultures of hCMEC/D3 cells and astrocytes on fibronectin-coated Rinzl at 1°C/min to –40°C before plunge into, and storage in liquid nitrogen yields monolayers with > 90% membrane integrity even after post-thaw overnight incubation. However, the metabolic activity of cryopreserved monolayers was lower relative to the unfrozen control, both when assessed immediately or after extended post-thaw incubation. Interestingly, co-cultures of hCMEC/D3 cells and astrocytes led to the formation of spherical-shaped aggregates, with dead cells appearing inside the spheroids, a behavior retained after cryopreservation. This is consistent with observations that the outer layers of a spheroid, which are highly exposed to the medium, are mainly comprised of viable cells, whereas the core cells which receive less oxygen, growth factors, and nutrients from the medium tend to be quiescent, necrotic, or apoptotic [81].

While only recently applied to endothelial cells by our group [37, 38, 46], the combination of DMSO and HES has been shown to synergistically improve cell viability following cryopreservation in many other cell types [82–84]. It has also been applied in the cryopreservation of skin fibroblast and keratinocyte cell lines in suspension and monolayers [85]. In that work, the authors showed that 5% DMSO and 5% HES in the presence of 90% fetal calf serum resulted in only about 50% viability post-thaw [85]. Similarly, using an improved media formulation (TiProtec, which proved favourable for cold storage of blood vessels) in the cryopreservation of porcine aortic endothelial cell monolayers enhanced mitochondrial integrity and yielded only 50% viable cells [86]. Addition of antifreeze proteins to 10% DMSO and slow cooling of A549 epithelial cell line monolayers improved post-thaw recovery from 25% to 60% [87, 88]. Directional freezing, in place of our controlled ice nucleation step, has been applied in the cryopreservation of epithelial cell line monolayers on glass coverslips. Gradual cooling at 1.2°C/min to –20°C, then at 0.5°C/min to, and storage in –80°C for 24 h resulted in 88% survival [89]; however, unlike our study viability was not examined after storage in liquid nitrogen.

## Conclusions

We describe here improved cryopreservation protocols for hCMEC/D3 cells and astrocytes in suspension that yielded ~90% post-thaw viability, and a newly established protocol for cryopreservation of endothelial monolayers applied to hCMEC/D3 and astrocyte single cultures and co-cultures. Using membrane integrity as a measure of cell viability, we showed that: *i*) for hCMEC/D3 cells in suspension the combination of 5% DMSO and 6% HES provides comparable cryoprotective effect as the manufacturer recommended combination of 5% DMSO and 95% FBS; *ii*) for astrocytes in suspension, 5% DMSO and 6% HES offered better cryoprotection than 10% DMSO; and *iii)* hCMEC/D3 and astrocyte single cultures or co-cultures can be cryopreserved as monolayers on fibronectin-coated Rinzl coverslips by cooling at 1°C/min to –40°C in the presence of 5% DMSO, 6% HES, and 2% chondroitin sulfate before plunging into, and storage in liquid nitrogen. Functional activity of cryopreserved hCMEC/D3 cells in suspension was demonstrated by their ability to form capillary networks in Matrigel, mimicking angiogenesis *in vivo*, and the expression of the tight junction protein ZO-1. Rinzl, with a coefficient of linear thermal expansion matched to ice, is a better cell culture surface for the cryopreservation of hCMEC/D3 monolayers than glass with a lower coefficient of linear thermal expansion or Thermanox with a higher coefficient of linear thermal expansion. The expression of junction proteins claudin-5 and ZO-1 in hCMEC/D3 monolayers that had been cryopreserved was similar to that of fresh unfrozen controls. The membrane integrity and the metabolic ability of cryopreserved monolayers of hCMEC/D3 and astrocyte single cultures or co-cultures were maintained following extended post-thaw incubation. Better methods for cryopreserving hCMECs/D3 cells and astrocytes in suspension and monolayers could expand their availability and accessibility for research on disease modeling, drug screening, and targeted therapy pertaining to the blood–brain barrier.

## Supporting information

S1 File (PDF)
