# Supplementary material for: Cryopreservation of human cerebral microvascular endothelial cells and astrocytes in suspension and monolayers
Source: PLoS One. 2021 Apr 14;16(4):e0249814. doi: 10.1371/journal.pone.0249814 (PMC8046249; doi:10.1371/journal.pone.0249814)
Supplement: S1 File — (PDF) [file pone.0249814.s001.pdf]

Supplementary Information for: "Cryopreservation of human cerebral microvascular endothelial cells and astrocytes in suspension and monolayers"

Leah A. Marquez-Curtis[1,2], Reid Bokenfohr[3], Locksley E. McGann[2], Janet A. W. Elliott[1,2]

1 Department of Chemical and Materials Engineering, University of Alberta, Edmonton, AB, Canada,
2 Department of Laboratory Medicine and Pathology, University of Alberta, Edmonton, AB, Canada,
3 Department of Medicine, University of Alberta, Edmonton, AB, Canada

The following tables contain the numerical values of data in the figures.

Table S1. Numerical values for data in Fig 1A: Doubling time for hCMEC/D3 cells grown in rat collagen-treated Falcon flasks *vs*. Corning CellBIND flasks

Doubling time (in days) of hCMEC/D3 in rat-collagen treated Falcon flasks *vs*. CellBIND flasks							
Passage number	31	32	33	34	Mean	SD	SEM
Rat collagen-treated Falcon flasks	1.44	1.209	1.399	1.145	1.29825	0.143405	0.071702
CellBIND flasks	1.303	1.237	1.598	1.193	1.33275	0.18252	0.09126

Table S2. Numerical values for data in Fig 1B: % membrane integrity (MI) of hCMEC/D3 cells in suspension subjected to graded freezing in the absence of cryoprotectants

Exp. Temp (° C)	% MI (CellBIND)		% MI (Collagen-treated flasks)		SEM (CellBIND)		SEM (Collagen-treated flasks)	
	Direct-thaw	Plunge-thaw	Direct-thaw	Plunge-thaw	Direct-thaw	Plunge-thaw	Direct-thaw	Plunge-thaw
0	98.33333	0.0267	98.03	0.207	0.273	0.027	0.433	0.098
−10	94.13333	1.4	93.07	0.483	1.220	0.551	1.309	0.3087
−20	62.7	11	60.77	13.47	3.756	1.350	7.452	4.0729
−30	25.3	11.533	23.07	10.43	4.748	1.746	5.398	1.8801
−40	8.5	10.6	7.633	7.0	0.404	2.411	2.567	2.5775

Table S3. Numerical values for data in Fig 2A: % membrane integrity of hCMEC/D3 cells in suspension subjected to graded freezing in the presence of 5% DMSO plus 6% HES

Exp. Temp (° C)	Mean % Membrane Integrity					
	5% DMSO+6% HES (washed)		SEM	n = 3	SD (PT)	
	Direct-thaw	Plunge-thaw	Direct-thaw	Plunge-thaw		p-value relative to –10 °C
0	96.93333	4	0.2000302	0.150023		
–10	96.3	72.96667	0.3500529	1.450219	2.51	
–20	96.5	80.66667	0.2000302	0.600091		
–30	95.86667	90.16667	0.0500076	0.500076	0.87	0.0004
–40	95.26667	91.26667	0.450068	0.850128	1.47	0.0004

Exp. Temp (° C)	Mean % Membrane Integrity					
	5% DMSO+6% HES (no wash)		SEM	n = 3	SD (PT)	
	Direct-thaw	Plunge-thaw (PT)	Direct-thaw	Plunge-thaw		p-value relative to –10 °C
0	98.5	5.1	0.152757	0.100003		
–10	95.43333	74.6	0.185598	4.2029	7.28	
–20	96.33333	82.86667	0.290602	4.410849		
–30	96	92.1	0.400012	0.776768	1.35	0.015
–40	96.03333	92.76667	0.554794	0.952802	1.65	0.014

Table S4. Numerical values for data in Fig 2B: % membrane integrity of hCMEC/D3 cells in suspension subjected to graded freezing in the presence of 5% DMSO plus 95% fetal bovine serum (FBS)

Exp. Temp (° C)	Mean % Membrane Integrity					
	5% DMSO+95% FBS (washed)		SEM	n = 3	SD (PT)	
	Direct-thaw	Plunge-thaw (PT)	Direct-thaw	Plunge-thaw		p-value relative to –10 °C
0	98.63333	17.26667	0.133337	3.735228		
–10	97.83333	83.26667	0.23334	0.52389	0.907	
–20	97.86667	86.86667	0.120189	0.497787		
–30	97.7	89.13333	0.057737	2.421044	4.19	0.08
–40	96.7	90.2	0.608294	0.65066	1.127	0.0012

Table S5. Numerical values for data in Fig 4B: Quantification of mean total length of tube formation of unfrozen hCMEC/D3 cells in suspension seeded on Matrigel as a function of time (hours after seeding)

Number of hours after seeding	1	2	3	4	5	6	8	24
	6779	5541	18413	19083	16844	15361	13180	7456
	5257	8001	21005	15779	15474	15869	14973	6956
	8186	9967	9923	16842	16851	17148	6769	6956
	5232	10165	9783	16098	14290	9271	6635	6365
	6745	7677	11115	13523	17156	4757	4417	3489
	6319	8791	8354	12500	14379	7262	6104	3489
	8224	8537	14034	12083	10032	6980		3683
	7118	9614	13138	11362	12262			3798
		8824	11138	14762	12484			4599
		10357	12475	14385	14090			2722
				12349	12389			3611
				12156	12729			3363
								4398
Mean	6732.5	8747.4	12937.8	14243.5	14081.7	10949.7	8679.67	4683.46
SD	1139.41	1448.17	3987.88	2349.76	2211.13	5043.2	4301.04	1640.92
SEM	402.902	457.9913	1261.188	678.3381	638.3169	1906.115	1755.527	455.8123

Table S6. Numerical values for data in Fig 4D: Comparison of mean tube length of unfrozen or cryopreserved hCMEC/D3 cells in suspension 4 h after seeding on Matrigel

SUMMARY of TUBE FORMATION (Fresh *vs.* cryopreserved in 5% DMSO+6% HES)			
FRESH		CRYOPRESERVED	
15/03/2019	15779	01/05/2019	12052
	16842		9756
	19083		13122
21/03/2019	16098		13752
	13523		15997
	12500		10351
	12083	03/05/2019	12919
	11362		16888
23/04/2019	14762		18630
	14385		11467
	12349		13543
	12156		15388
06/05/2019	12228	06/05/2019	12829
	13770		10537

	11129		10575
	11806		14872
	11634		13371
	10654		12506
n = 18	13452.39	Mean	13253.06
SD	2289.256	SD	2399.497
SEM	539.9189	SEM	565.9191
		Mean tube length	SEM
unfrozen control		13452	540
cryopreserved		13253	566
n = 18		p = 0.8	

Table S7. Numerical values for data in Fig 5. Comparison of membrane integrity of astrocytes in suspension (unfrozen *vs*. cryopreserved in the presence of 10% DMSO *vs*. 5% DMSO plus 6% HES)

Condition	% Membrane Integrity				Mean (n = 4)	SD	SEM	p-value relative to control	p-value between CPA
Unfrozen control	88.2	93.0	92.4	92.2	91.45	2.193	1.097		
DMSO	80.4	81.8	80.0	78.2	80.1	1.483	0.742	0.0001	
DMSO+HES	89.1	85.7	92.1	92.2	89.775	3.074	1.537	0.41	0.001

Table S8. Numerical values for data in Fig 6. Comparison of live (unfrozen) control and cryopreserved monolayers on glass, Rinzl and Thermanox in terms of percent membrane integrity, absolute viability, and metabolic activity as assessed by AlamarBlue reduction assay

Rinzl	Mean		SEM	
	% Membrane integrity	Absolute viability	% Membrane integrity	Absolute viability
Live control	98.84562	98.9276	0.395	0.319
–40 °C PT (same day)	86.4537	88.2503	0.746	7.543
–40 °C PT (next day)	76.92296	81.287	3.967	9.79

Glass	Mean		SD		n	SEM	
	% Membrane Integrity	Absolute viability	% Membrane integrity	Absolute viability		% Membrane integrity	Absolute viability
Live control	98.3125	98.1446	1.756	6.08	5	0.78	2.72
–40 °C PT (same day)	2.9073	2.12787	2.797	1.62	6	1.14	0.662
–40 °C PT (next day)	4.33125	1.95055	2.890	1.107	6	1.18	0.452

Thermanox	Mean % Membrane Integrity	Mean Absolute Viability	n	SEM (% Membrane Integrity)	SEM (Absolute Viability)
Live control	97.32	97.32	5	0.653778	4.778367
–40 °C PT (same day)	50.7	55.85	6	8.606642	12.06798
–40 °C PT (next day)	75.75	73.05	4	9.061411	9.189623

AlamarBlue Reduction Assay						
	Glass		Rinzl		Thermanox	
	Mean	SEM	Mean	SEM	Mean	SEM
Live control	65.5	21	65.2497	16.31409	52.89882	2.810827
–40 °C PT (same day)	8.4	2.6	50.64295	8.015376	10.68742	4.83385
–40 °C PT (next day)	16.8	4	56.33803	4.362622	23.18458	2.844181

Table S9. Numerical values for data in Fig 8B. Percent membrane integrity and absolute viability of unfrozen (live control) and cryopreserved astrocyte monolayers on Rinzl

	Mean			SEM	
	% Membrane Integrity	Absolute Viability	n	% Membrane Integrity	Absolute Viability
Live control same day	96.36667	96.53333	3	1.669713	1.659701
–40 °C PT same day	86.48	52.62	5	2.076936	12.34053
Live control next day	95.83333	95.93333	3	0.705554	0.775336
–40 °C PT next day	94.7	66.275	4	1.334166	13.47747

Table S10. Numerical values for data in Fig 8C. AlamarBlue reduction activity of unfrozen (live control) and cryopreserved astrocyte monolayers on Rinzl

Alamar Blue reduction (corrected for no cell/media only control)							
					Mean	SD	SEM
Live control same day	57.1	59.2	73.5	93.2	70.75	16.64622	8.32311
–40 °C PT same day	36.2	41.7	42.7	54.1	43.675	7.514597	3.757298
Live control next day	55.4	68.9	61.8	68.8	63.725	6.469093	3.234547
–40 °C PT next day	45.9	37.5	46.5	72.3	50.55	15.07083	7.535416

Table S11. Numerical values for data in Fig 9B. Percent membrane integrity and absolute viability of unfrozen (live control) and cryopreserved co-cultures of hCMEC and astrocyte monolayers on Rinzl

	Mean			SEM		p-value of % Membrane Integrity
	% Membrane Integrity	Absolute viability	n	% Membrane Integrity	Absolute viability	
Live control same day	98.13	98.07	3	0.317989	0.333343	
–40 °C PT same day	90.8	86.425	4	2.27413	6.080484	0.024
Live control next day	98.325	98.35	4	0.35678	0.366288	
–40 °C PT next day	91.15	75.175	4	0.98192	6.354182	0.0005

Table S12. Numerical values for data in Fig 9C. AlamarBlue reduction activity of unfrozen (live control) and cryopreserved co-cultures of hCMEC and astrocyte monolayers on Rinzl

					Mean	SD	SEM		
Live control same day	35.2	61.6	70.7	47.8	53.825	15.58212	7.791061	p-value relative to live	p-value of cryo next day
–40 °C PT same day	33.2	27.7	25.5	31.1	29.375	3.436447	1.718223	0.022	
Live control next day		66.27	65.14	65.8	65.73667	0.567656	0.327746		
–40 °C PT next day	41.13	40.8	53.49	40.68	44.025	6.312868	3.156434	0.001	0.0065
